# Stable Protein‐Based G‐Quadruplex‐Derived Supramolecular Bioinks as Tunable ECM‐Mimetic Constructs Assembled by Combining Non‐Covalent and Covalent Strategies

**DOI:** 10.1002/adma.202521259

**Published:** 2026-04-08

**Authors:** Vera Sousa, Rita Sobreiro‐Almeida, Bart W. L. van den Bersselaar, E. W. Meijer, Ghislaine Vantomme, João Borges, João F. Mano

**Affiliations:** ^1^ CICECO – Aveiro Institute of Materials Department of Chemistry University of Aveiro Campus Universitário de Santiago Aveiro Portugal; ^2^ Institute for Complex Molecular Systems Eindhoven University of Technology Eindhoven The Netherlands; ^3^ Department of Chemical Engineering and Chemistry Laboratory of Macromolecular and Organic Chemistry Eindhoven University of Technology Eindhoven The Netherlands

**Keywords:** 3D bioprinting, covalent reinforcement, G‐quadruplex hydrogels, macromolecular crowding, supramolecular self‐assembly

## Abstract

G‐quadruplex hydrogels hold great promise for biofabrication owing to their dynamic supramolecular nature, provided their inherent instability under physiological conditions is overcome. Here, a bioinspired strategy that synergistically combines supramolecular self‐assembly, under macromolecular crowding conditions, with in‐bath enzymatic covalent crosslinking was employed to create stable, protein‐based G‐quadruplex‐derived hydrogels. Mimicking the crowded intracellular milieu, the addition of Ficoll enhances G‐quadruplex stability and tunes the rheological behavior, while transglutaminase‐mediated crosslinking reinforces the network, preserving its structural integrity over extended periods. This combined approach yields printable bioinks with optimal viscosity, yield stress, and shear‐thinning properties, enabling the fabrication of complex, multilayered 3D constructs that support enhanced cell viability and proliferation within an extracellular matrix (ECM)‐mimetic fibrillar environment. Moreover, the modulation of the crosslinking density allows controlling cellular responses, offering a versatile platform for tailoring the biomechanical microenvironment. This study establishes a new class of hybrid G‐quadruplex hydrogel bioinks, exhibiting unprecedented stability under physiological conditions, biofunctionality, and off‐the‐shelf availability, unlocking their potential for advanced tissue engineering and regenerative medicine strategies.

## Graphical abstract

Harnessing the synergistic interplay of supramolecular self‐assembly, under macromolecular crowding conditions, and enzymatic‐mediated covalent crosslinking toward a stable protein‐based G‐quadruplex‐derived supramolecular bioink. This bioinspired strategy enables the biofabrication of complex and tunable ECM‐mimetic constructs, providing a platform with enhanced biofunctionality. This approach establishes a new paradigm in biofabrication, bridging supramolecular chemistry, covalent crosslinking and bioinspired design to create tissue‐like constructs.

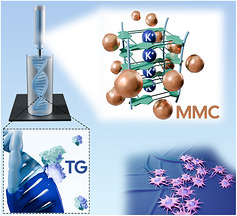

## 1 Introduction

Supramolecular self‐assembly is a ubiquitous process in biological systems, relying on the spontaneous organization of molecular building blocks through non‐covalent forces into self‐assembled hierarchical structures [1, 2, 3]. G‐quadruplexes, unique non‐canonical four‐stranded structures found in guanine‐rich DNA or RNA sequences, have garnered considerable attention in the biomaterials field owing to their unique self‐assembled structures [4], dynamic nature [5], and multiple biological roles [6, 7]. Frequently, boronic acid derivatives are coupled to guanosine (G) moieties via dynamic boronate ester bonds to enhance the self‐assembly‐driven hydrogelation, acting as low‐molecular‐weight gelators [8, 9, 10, 11]. Furthermore, these boronic acid units can be covalently conjugated to natural polysaccharides either through stable amide bonds [9, 12] or via dynamic boronate ester bonds [13], resulting in the formation of G‐quadruplex hydrogels with improved viscoelastic properties. In fact, it is noteworthy that formylphenylboronic acid derivatives can form both dynamic imine and boronate ester bonds with primary amines (─NH_2_) and *cis*‐1,2‐diols, respectively [14, 15, 16, 17, 18, 19, 20, 21, 22]. More specifically, through their aldehyde reactive group, these boronic acid derivatives can generate imino‐boronate bonds with NH_2_‐containing macromolecules, particularly with aminated synthetic polymers [15, 17, 23], peptides [18, 19], and pseudopeptides [24].

G‐quadruplex hydrogels have garnered considerable attention for a wide range of applications, such as drug delivery [15, 17, 19, 25, 26], cell culture [9, 27], tissue engineering [4, 9], sensing and imaging [28], or as contaminant adsorbents [29], and emulsion stabilizers [30]. Despite their attractive features, G‐quadruplex hydrogels suffer from limited stability under physiological conditions, undergoing irreversible disassembly mainly due to their hydrophilic nature [9, 12, 31], which remains as a major bottleneck for biomedical applications. Benchtop stability is often used as a measure, and it is typically assessed without immersing the hydrogels in aqueous solutions, failing in reproducing their performance under complex biological environments. As such, hydrogels, which are reported to be stable under dry conditions for weeks [9] or even months [32], end up being dissolved within 12 h [9] or 3 days [32] when incubated in culture medium. In both cases, their long‐term stability in cell culture, which is essential for bioprinting applications, is severely compromised. Although this instability is an intrinsic feature of G‐quadruplex hydrogels, these supramolecular structures are easily formed and highly stable in DNA [33]. Among other factors, the heavily crowded intracellular environment, where water activity decreases and hydration becomes unfavorable [34, 35], plays a crucial role in enhancing the stability of G‐quadruplex structures, a behavior that can be mimicked by (macro)molecular crowders such as poly(ethylene glycol) (PEG) and glycerol [34, 36, 37, 38]. Within DNA, the G‐quadruplex formation is driven by Hoogsteen base‐pairing and competes with the conventional duplex hybridization via Watson‐Crick base‐pairing. In fact, it has been shown that, by destabilizing the duplex and stabilizing the quadruplex, macromolecular crowding affects the quadruplex‐duplex equilibrium in living cells [39]. This phenomenon relies on the fact that duplex formation requires hydration due to the presence of additional water‐binding sites. On the contrary, in the G‐quadruplex structure, where most of the hydration sites are occupied by the Hoogsteen‐type hydrogen bonding interactions, the presence of water disturbs the supramolecular self‐assembly [34, 39]. Despite this behavior, the use of macromolecular crowders (MMCs) as a bioinspired strategy to enhance the long‐term stability of G‐quadruplex hydrogels under physiological conditions remains unexplored.

Hydrogels are particularly well‐suited for tissue engineering due to their close similarity to the native ECM of tissues [40], providing essential structural and mechanical support to cells, thus facilitating cell adhesion, migration, and matrix remodeling within a 3D environment, which is crucial for the proper development of functional tissues [40, 41]. Conventional hydrogels, which usually rely on 3D polymeric networks stabilized by covalent bonds, denote excellent mechanical strength and stability, and high water content [42, 43]. However, robust covalent crosslinking can turn these networks into brittle formulations, deprived of self‐healing characteristics, which limit their potential application as injectable or printable materials [44]. Supramolecular chemistry can mitigate this issue by enabling rapid bond reformation after damage, thereby restoring the network and its viscoelastic properties [44, 45]. By offering a superior level of dynamicity and adaptability, supramolecular hydrogels emerge as ideal matrices to mimic the structural, mechanical, and biochemical complexity of native tissues, which is a central challenge in regenerative medicine and biofabrication. In fact, researchers have demonstrated that the printability of supramolecular inks — based on benzene‐1,3,5‐tricarboxamide [46], peptide amphiphiles (PAs) [47], or natural polymers [48] — can be precisely modulated by tailoring the molecular design of the building blocks [46] or by adjusting the intrinsic self‐assembly conditions such as pH and salt concentration [47, 48]. These strategies enable fine‐tuning of key parameters, including ink viscosity [47] and the shape‐fidelity of the printed constructs [46, 48]. Furthermore, aiming to provide a biologically instructive milieu, supramolecular inks have been engineered by leveraging the co‐assembly of PAs with whole blood, thus incorporating crucial biochemical and structural cues of the regenerative hematoma [49]. Despite their remarkable tunability, these inks face challenges in the fabrication of complex and multilayered 3D structures and, in certain cases, their self‐assembly conditions can restrict cell encapsulation. G‐quadruplex hydrogels, as DNA‐inspired supramolecular assemblies, harness the simplicity of natural molecular architectures to combine biodegradability and multifunctionality with scalable and cost‐effective fabrication [4]. However, their potential as (bio)inks remains largely unexplored, with only a few reports emerging in recent years [50, 51, 52]. This can be ascribed to intrinsic limitations, ranging from filament spreading that compromises shape‐fidelity, limited post‐printing crosslinking strategies, instability under physiological environments, and lack of cell‐instructive cues. In nature, covalently reinforced supramolecular networks are ubiquitous [53], from collagen [54] and fibrous elastin [55] to human insulin [56] and fibrin clots [57]. These naturally occurring hybrid assemblies serve as an extraordinary source of inspiration for designing exquisite (bio)inks that combine structural stability with enhanced 3D‐printing performance [58, 59]. Nevertheless, such a hybrid approach has not yet been explored with G‐quadruplex hydrogels.

Herein, a novel and stable protein‐based G‐quadruplex‐derived supramolecular hydrogel was successfully produced by multicomponent self‐assembly of G, 2‐formylphenylboronic acid (2‐FPBA), and gelatin in the presence of potassium cations (K^+^). By leveraging the bioinspired combination of transglutaminase (TG)‐mediated covalent crosslinking in the support bath and the addition of Ficoll as a MMC to the supramolecular hydrogel formulation, we developed a bioink exhibiting enhanced stability, superior shape‐fidelity, and improved structural complexity, being suitable for encapsulating primary human‐derived cells. In contrast to other hybrid systems, this synergistic approach, reported here for the first time, requires no redesign of the building blocks while emulating the heavily crowded cellular microenvironment. The suitable and tunable rheological properties, along with the feasibility of printing complex 3D structures denoting enhanced biological performance, unlock endless possibilities for personalized tissue engineering and regenerative medicine strategies, including on‐demand therapeutic solutions for a variety of targeted tissues.

## 2 Results and Discussion

### 2.1 Formation of the G‐Quadruplex Supramolecular Hydrogels

**SCHEME 1:**
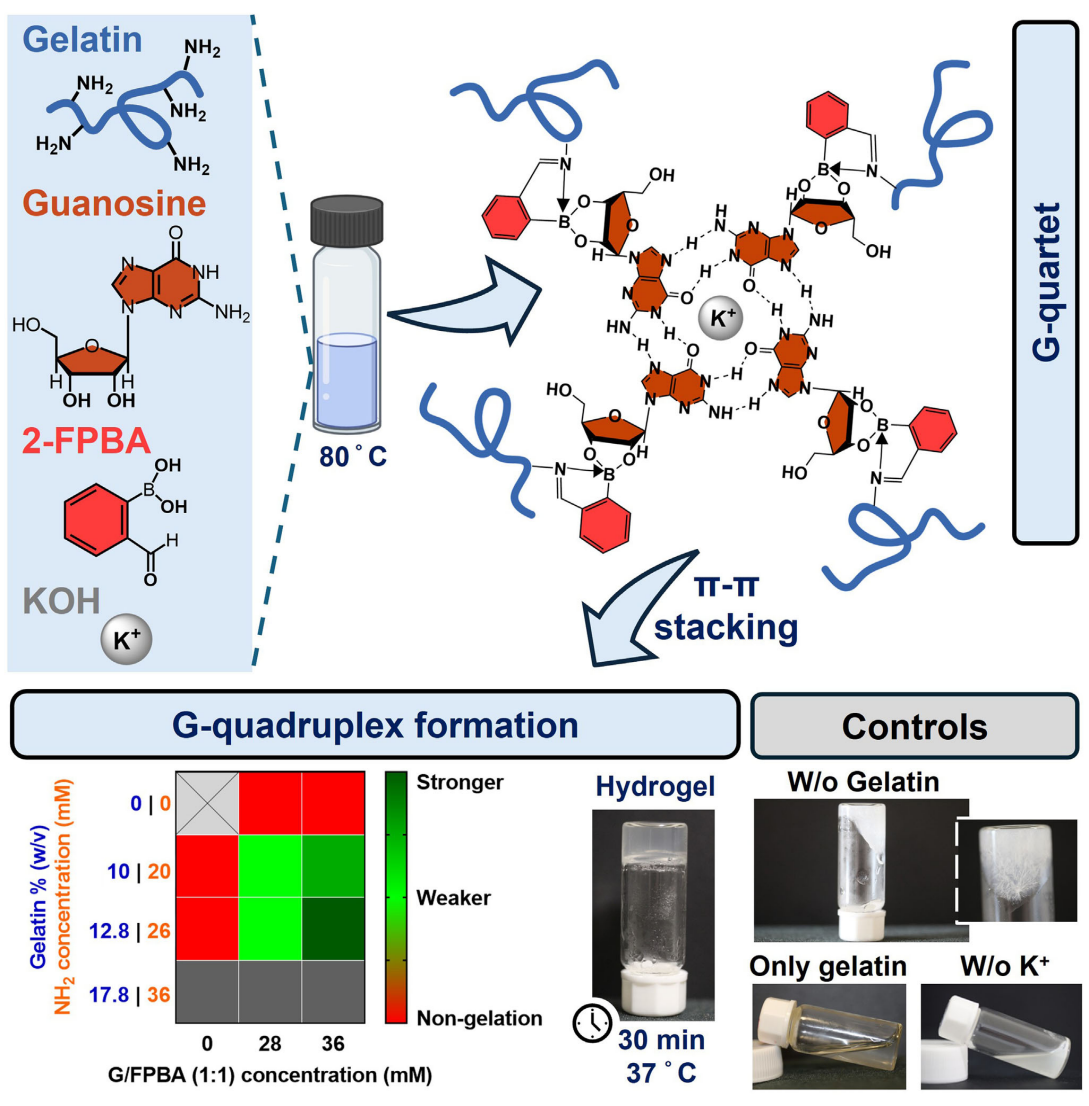
Schematic illustration of the building blocks and self‐assembly process behind the formation of gelatin‐based G‐quadruplex‐derived supramolecular hydrogels, prepared by heating (80°C, 30 min) and cooling (37°C, 30 min) a KOH aqueous solution containing gelatin, G, and 2‐FPBA. A heat map summarizing all tested conditions at a constant KOH concentration of 66 mM is presented, with dark grey indicating conditions where gelatin alone formed a gel at 37°C, which were therefore excluded from further studies. Digital images of the controls, highlighted in red on the heat map, are also included.

The gelatin‐based G‐quadruplex‐derived supramolecular hydrogel was assembled by mixing individual components in specific ratios. G‐quartets (G4), the building blocks of G‐quadruplexes, are formed by hydrogen bonding interactions between four G in a square planar arrangement and stabilized by K^+^ ions. These hydrogen‐bonded G4 monomers are linked via dynamic cyclic boronate ester bonds to 2‐FPBA. The latter acts as an anchor for the bioconjugation of the G4 supramolecular entities to gelatin through imine bonds formed between its aldehyde moiety and the free ─NH_2_ groups of the protein, facilitating the assembly of the imino‐boronate ester complex. Furthermore, higher‐order, protein‐based G‐quadruplex supramolecular structures are assembled through the *π*–*π* stacking of the G4 monomers, ultimately resulting in the formation of a hydrogel after 30 min of incubation at physiological temperature (Scheme 1). To gain deeper insights into the influence of the free ─NH_2_ groups on the G‐quadruplex hydrogel formation, a 2,4,6‐trinitrobenzenesulfonic acid (TNBS) assay was conducted on gelatin (Figure S1). As shown in the heat map (Scheme 1), a stronger and self‐supporting hydrogel is formed by increasing the concentration of 2‐FPBA and G to 36 mM, as well as the ─NH_2_ content to 26 mM. In contrast, the control experiments depicted in Scheme 1 reveal that gelatin alone, at 37°C, remains completely liquid.

Moreover, hydrogelation fails in the absence of either gelatin or K^+^, leading to guanosine precipitation. These results emphasize the critical role played by all hydrogel components, resulting in a multicomponent self‐assembly process to successfully achieve gelation. The developed hydrogel was further analyzed by ^1^H NMR spectroscopy at 37°C to elucidate the multicomponent reaction‐driven gelation process (Figure S2a). ^1^H NMR spectra confirmed the formation of dynamic boronate ester and imine bonds, as evidenced by the peaks at δ = 5.55 ppm (H_a_) and δ = 8.66 ppm (H_c_), respectively [9, 17, 18]. Additional peaks were observed at δ = 6.05 ppm (H_b_) and δ = 10.12 ppm (H_d_), corresponding to unreacted G and unreacted 2‐FPBA, respectively. Furthermore, the integration ratio between the reacted and unreacted species revealed that 11% of boronate ester bonds and 26% of imine bonds were formed within the hydrogel network. The ^1^H NMR spectrum was also recorded at 25°C for comparison (Figure S2b). In contrast to the behavior observed at 37°C, only the peak assigned to unreacted G (H_b_) was detected (detailed discussion provided in the Supporting Information).

To complement these findings, the formulated hydrogel was further characterized by ATR‐FTIR spectroscopy (Figure S3a). The ATR‐FTIR spectrum of the G‐quadruplex hydrogel revealed traces of the vibration band associated with G‐free diols (*ν*_C−OH_ = 1078 cm^−1^) [9], along with the absence of the characteristic vibration band of free boronic acid diols (ν_B−OH_ = 1352 cm^−1^) [9]. These findings suggest the formation of boronate ester bonds, resulting from the reaction between the 2‐FPBA and the *cis*‐1,2‐diols of G, which was further confirmed by the appearance of a new vibration band at 1110 cm^−1^ (ν_B−OC_) [17, 18, 19]. Furthermore, the appearance of the stretching vibration at 1694 cm^−1^ (ν_C = N_) [17], together with the absence of the ν_C = O_ band at 1658 cm^−1^, which is characteristic of the aldehyde group in 2‐FPBA, suggests the formation of imine bonds within the hydrogel network. Interestingly, no C═N bond formation was detected in a G‐free mixture containing 2‐FPBA, gelatin, and K^+^, indicating that the formation of imine and boronate ester bonds occurs synergistically (Figure S3b) [23]. This can be explained by the nitrogen atom of the imine bond donating its lone pair of electrons to the boron center through a dative B─N bond, thereby facilitating the assembly of the imino‐boronate ester complex [19, 23, 60, 61]. Furthermore, as expected, the ν_B−OC_ vibration band was not observed due to the absence of G‐free diols in the mixture.

### 2.2 Role of Macromolecular Crowding on G‐Quadruplex Self‐Assembly

**FIGURE 1:**
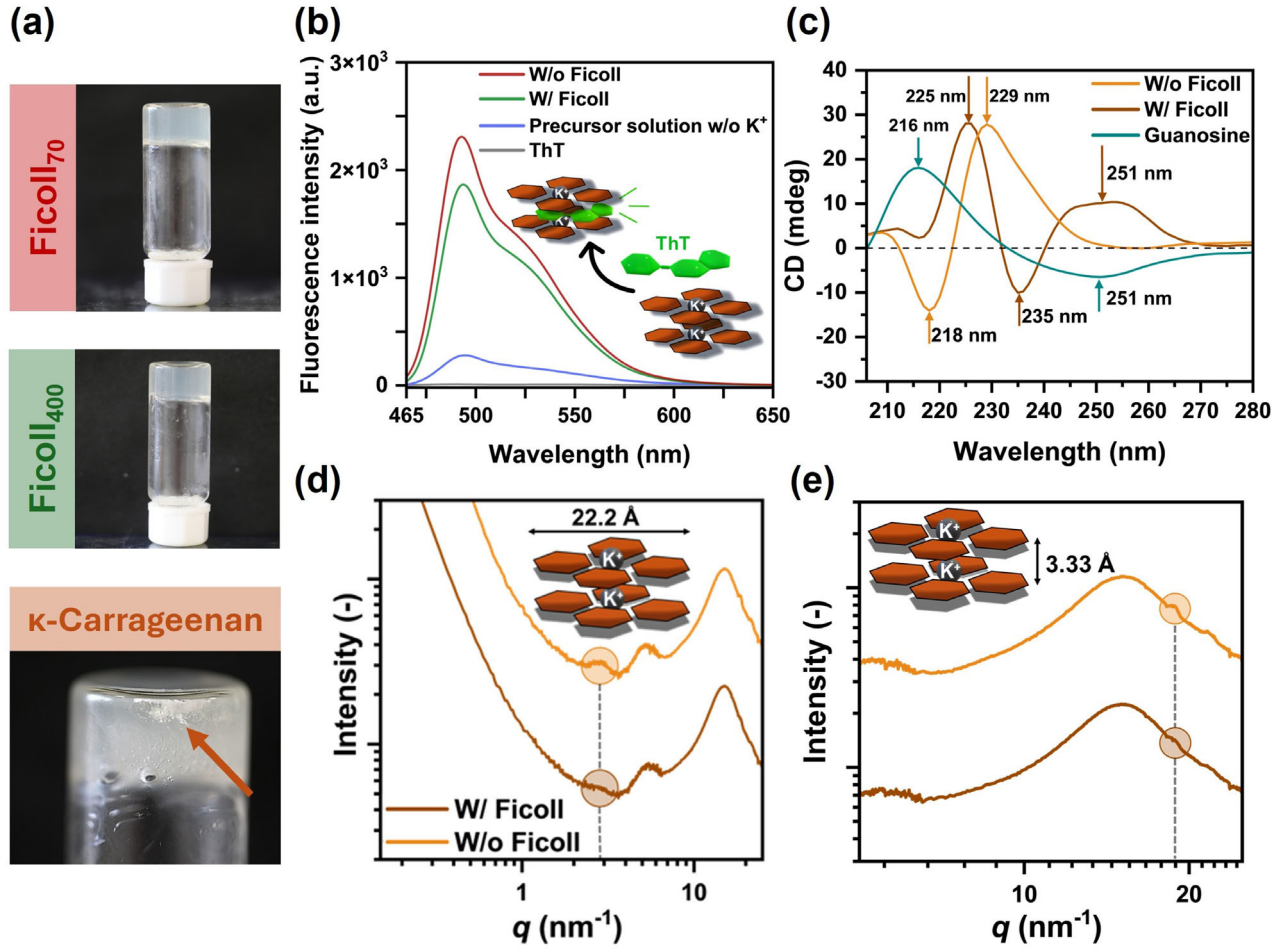
Role of macromolecular crowding on G‐quadruplex self‐assembly: (a) photographs of gelatin‐based G‐quadruplex‐derived hydrogels with different MMCs; (b) ThT fluorescence assays of the hydrogels in the presence and absence of MMC; (c) circular dichroism spectra in the diluted state; (d) medium and (e) wide‐angle X‐ray scattering patterns of the freeze‐dried hydrogels.

The stability of the G‐quadruplex structures increases significantly in macromolecular crowding environments, since the presence of MMCs reduces the quantity and activity of water molecules surrounding the structure [34]. Taking inspiration from this naturally occurring biological process within the cellular microenvironment, different MMCs were incorporated into the network to identify the most effective one for stabilizing the G‐quadruplex hydrogel. Accordingly, sulfated and neutral polysaccharides, such as κ‐carrageenan and Ficoll (70 and 400 kDa), respectively, were tested as MMCs, as they are commonly used in cell culture for promoting the ECM deposition and alignment [62, 63, 64, 65]. As shown in Figure 1a, the presence of Ficoll does not affect hydrogel formation, whereas the incorporation of κ‐carrageenan results in the precipitation of G. This phenomenon can be attributed to the ability of κ‐carrageenan to hydrogelate in the presence of K^+^ cations, as it promotes cation‐dependent aggregation between sulfated carrageenan helices [66], thereby sequestering the K^+^ cations needed for the formation of the G‐quadruplex structure. As such, κ‐carrageenan was excluded from further studies. In addition, a G‐free control mixture was prepared to assess whether the additional crosslinking between 2‐FPBA and the MMC could occur (Figure S4a; detailed discussion provided in the Supporting Information). Furthermore, the effect of Ficoll's molecular weight on the stability of the G‐quadruplex hydrogels was assessed through degradation studies at 25 mg/mL, a concentration often selected to emulate the crowded extracellular space [63, 65]. As shown in Figure S4b, Ficoll with a higher molecular weight was identified as the most effective MMC due to the slower degradation profile observed for the respective hydrogel. Therefore, it was selected for further studies. However, it is noteworthy that, although it significantly improves the hydrogel stability, the presence of MMC alone was not enough to stabilize the hydrogel over an extended period, with complete degradation being observed after 12 h. This limited stability is not unprecedented and reflects a major challenge associated with G‐quadruplex‐based biomaterials. In previous studies, in vitro experiments had to be shortened due to hydrogel complete dissolution in the culture medium after 48 h [27]. Likewise, a study on G‐quadruplex‐based antiviral hydrogels reported complete degradation after just 8 h at 37°C [67].

To further investigate the influence of the MMC on the formation of the G‐quadruplex supramolecular structures, a Thioflavin T (ThT) fluorescence assay was performed (Figure 1b). This hydrophilic cationic dye enhances its fluorescence intensity upon selective binding to G‐quadruplex structures, making it a widely used method for monitoring G‐quadruplex formation within hydrogels [9, 17, 18, 19, 68]. The results reveal that, in the absence of MMC, the fluorescence intensity of the ThT‐loaded gelatin‐based G‐quadruplex hydrogel increased by ca. 211‐fold when compared to the very weak fluorescence emission peak of the ThT solution alone (Figure 1b, red line). In contrast, although the addition of ThT to a precursor mixture lacking K^+^ resulted in a slight increase in the fluorescence intensity, it was negligible when compared to the pronounced fluorescence enhancement observed after ThT binding to the G‐quadruplex self‐assembled structures. Under macromolecular crowding conditions, the fluorescence intensity of the G‐quadruplex hydrogel increased significantly by ca. 171‐fold, but to a lesser extent than in the hydrogel without MMC (Figure 1b, green line). This difference might be due to the fact that macromolecular crowding environments can disturb the interaction between the G4 and their ligands, such as ThT [36, 69, 70], mainly due to the reduced water activity in crowded systems, which decreases the molecular diffusion rates and limits the effective binding of G4 ligands [36]. These results not only confirm the formation of G‐quadruplex self‐assembled structures within the hydrogel network but also highlight the impact of macromolecular crowding on the efficient binding of small ligands, such as ThT.

Circular dichroism (CD) spectroscopy was employed to further investigate the structural characteristics of the G‐quadruplex assemblies and to evaluate the influence of macromolecular crowding on their secondary structure [9, 71, 72, 73, 74, 75]. Typically, a positive band at ∼260 nm and a negative band at ∼240 nm are indicative of a parallel secondary structure, whereas two positive bands at ∼240 nm and ∼290 nm, along with a negative band at ∼260 nm, are characteristic of an antiparallel structure [9, 71, 72, 73, 74, 75]. A parallel G‐quadruplex structure is formed when all G within individual G4 are oriented in the same direction, specifically adopting the same glycosidic bond conformation (*anti‐anti‐anti‐anti* or *syn‐syn‐syn‐syn*). In contrast, an antiparallel G‐quadruplex structure is formed when the G4 comprise two guanosines in the *syn* conformation and another two in the *anti* conformation (*syn‐syn‐anti‐anti* or *syn‐anti‐syn‐anti*) [73]. As shown in Figure 1c, an aqueous G solution exhibits a positive band at 216 nm and a negative band at 251 nm. When compared to the G CD profile, the Ficoll‐free G‐quadruplex self‐assembled structure exhibited an inversion of chirality at 218 nm with negative ellipticity, followed by a positive band at 229 nm (Figure 1c, orange line).

Despite the observed blueshift in the characteristic bands of the G‐quadruplex structures, the overall CD spectrum suggests that the structure mostly adopts a parallel conformation, as previously reported [9]. In contrast, the sample containing Ficoll exhibited positive and negative bands at 225 and 235 nm, respectively, roughly opposite to the observed bands for the parallel secondary structure (Figure 1c, brown line). In addition, when compared to the G CD profile, an inversion of chirality was denoted at 251 nm, showcasing a positive band. This behavior indicates that the presence of Ficoll induces a conformational change from a parallel to an antiparallel secondary structure, which has been reported to be thermodynamically more stable [34, 76, 77, 78]. In fact, crowder‐induced modulation of G‐quadruplex conformation has been widely reported in the literature [36, 79, 80, 81, 82, 83], with PEG being frequently associated with the stabilization of the parallel conformation [36, 82, 83], whereas Ficoll and ethylene glycol have been shown to favor antiparallel structures [84, 85]. While these studies have focused mainly on oligonucleotides or human telomeric G‐quadruplex‐forming sequences, this work showcases the first report of such an effect in G‐quadruplexes assembled from G as a standalone building block. Furthermore, control samples including Ficoll, gelatin, and FPBA were also analyzed for comparison (Figure S5a). As expected, at 37 c°C, gelatin exhibited a random coil conformation [86], while the remaining controls were CD silent. It is well established that MMCs can influence the protein structure, folding, shape, and conformation, often promoting the transition of unfolded states to more compact structures [87, 88, 89, 90]. Having this in mind, the CD analysis of a mixture containing both gelatin and Ficoll was conducted, revealing that gelatin maintained its random coil configuration, thus proving its conformational stability under macromolecular crowding conditions (Figure S5a). These findings not only support previous studies indicating that Ficoll does not specifically interact with proteins [91, 92], but also validate Ficoll as a suitable crowder, as it does not interfere with the protein, enabling a reliable evaluation of macromolecular crowding effects on G‐quadruplex structures.

While the CD spectroscopy provided valuable insights into the conformational features of the G‐quadruplex assemblies, small‐angle X‐ray scattering (SAXS) was further employed to elucidate structural details at the molecular level, including the diameter of a single G4 monomer and the *π*–*π* stacking distance between two adjacent G4 entities. The medium/wide‐angle X‐ray scattering (MAXS/WAXS) patterns of the freeze‐dried hydrogels, both with and without Ficoll, showcased a broad peak at 2.83 nm^−^^1^ in the MAXS region, corresponding to a *d*‐spacing of 22.2 Å, which is consistent with the diameter of the G4 monomer (Figure 1d) [29]. In the WAXS region, both hydrogels exhibited a characteristic peak at 18.8 nm^−^^1^, which corresponds to a *d*‐spacing of 3.33 Å, attributed to the *π*–*π* stacking distance between two adjacent G4 monomers (Figure 1e) [9, 74, 93, 94]. In contrast, the MAXS/WAXS patterns of the control experiments did not contain peaks at 2.83 or 18.8 nm^−^^1^ (Figure S5b). In addition, the gelatin spectrum merely displayed a broad peak around 5.4 nm^−^^1^, corresponding to a disorder spacing of approximately 12 Å, assigned to the interhelical distance of gelatin [95, 96]. These results, along with the CD analysis, allow us to conclude that, despite the observed changes in the secondary structure, the diameter and *π*–*π* stacking distance of the G4 monomers remain unchanged, regardless of the presence of the MMC.

Z‐contrast (ZC)‐scanning transmission electron microscopy (STEM) mode images (Figure S5c), which are highly sensitive to the atomic number of elements [97], revealed that both G‐quadruplex samples, with and without Ficoll, exhibited fibrillar networks. However, the network became more densely packed and highly interconnected in the presence of MMC. In contrast, no fiber formation was found in the gelatin‐only control sample. These observations suggest that Ficoll, by reducing the available space in the system, facilitates the closer packing of fibers [38, 98, 99].

### 2.3 Synergistic Effect of Ficoll‐Induced Dehydration and Enzymatic‐Mediated Covalent Crosslinking on G‐Quadruplex Stability

**FIGURE 2:**
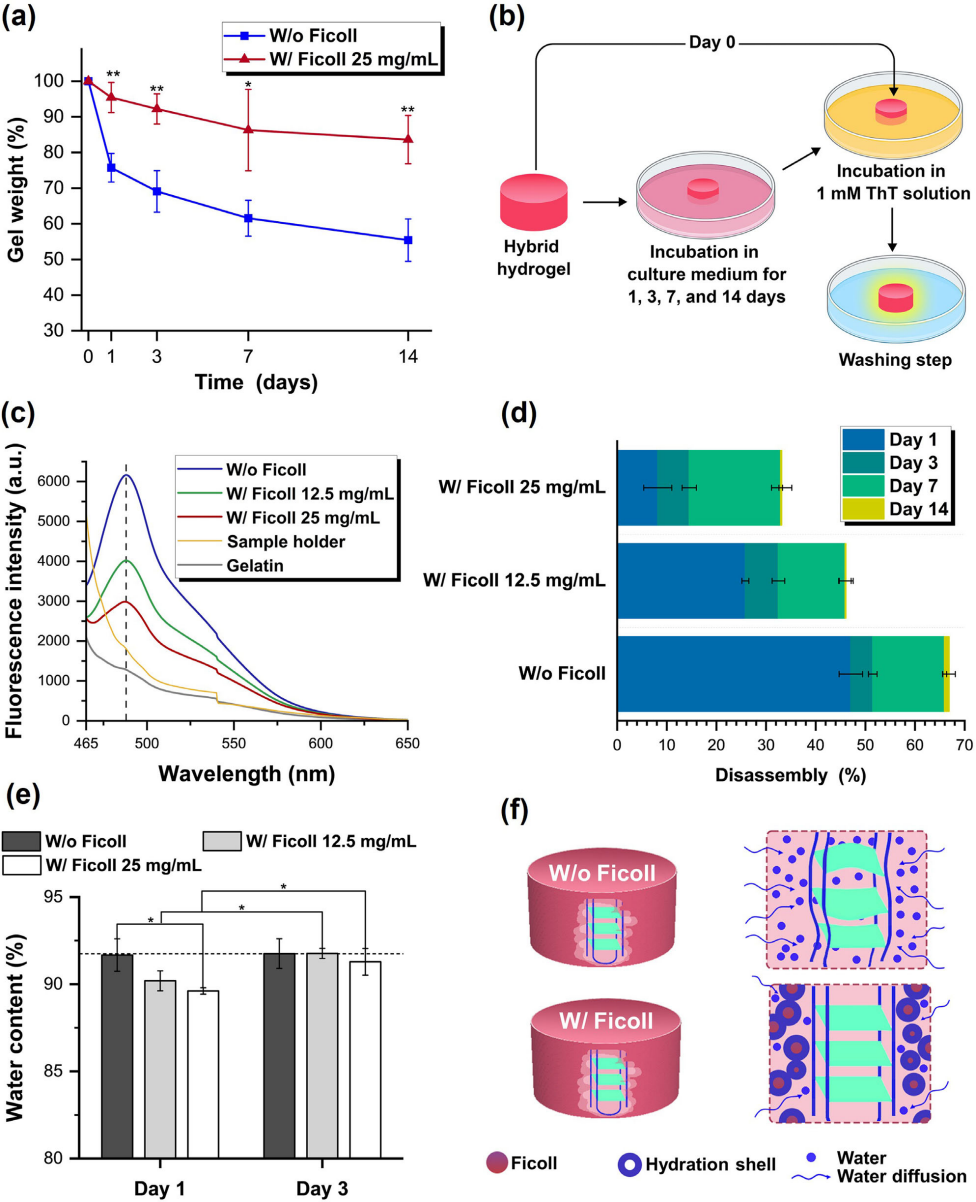
Synergistic effect of Ficoll‐induced dehydration and in‐bath enzymatic‐mediated crosslinking to enhance G‐quadruplex stability: (a) degradation studies (*N* = 3); (b) hybrid G‐quadruplex hydrogels were incubated with ThT to evaluate initial (day 0) and time‐dependent G‐quadruplex content after incubation in culture medium at 37°C; (c) fluorescence profile of all hydrogel formulations immediately after TG‐mediated crosslinking (day 0). Fluorescence spectra of gelatin and of the empty sample holder were also recorded for comparison, indicating the absence of the characteristic ThT emission peak (dashed line); (d) G‐quadruplex disassembly over time across all tested hybrid hydrogel formulations (*N* = 3); (e) water content evaluation of the hybrid G‐quadruplex hydrogels (*N* = 3); and (f) proposed mechanism illustrating how macromolecular crowding alters water content and its diffusion, contributing to G‐quadruplex stabilization. Statistical analysis was performed using an unpaired t‐test. Individual significance levels are indicated as follows: ^*^p < 0.05, ^**^p < 0.01.

As discussed previously, the presence of MMC alone was not sufficient to stabilize the hydrogels for extended periods. Inspired by biochemical processes that occur in the human body [57, 100, 101], an additional in‐bath covalent crosslinking was employed after the G‐quadruplex self‐assembly using 5% (w/v) of TG. This enzyme catalyzes the formation of isopeptide bonds between the γ‐carboxamide groups of glutamine and the ε‐amino groups of lysine, namely within the gelatin chains [102], and such bonds are known to be highly resistant to hydrolysis [103, 104, 105, 106], thus enhancing the construct's stability under physiological conditions, which is essential for biomedical applications. Thus, after combining both the physical and chemical crosslinking (hybrid hydrogels), degradation studies were again conducted to assess whether macromolecular crowding environments could significantly influence the stability of G‐quadruplex structures even after TG‐mediated crosslinking. As shown in Figure 2a, the hybrid hydrogel containing Ficoll lost only 16% of its weight after 14 days, whereas the Ficoll‐free hydrogel lost 45%. These results clearly demonstrate that the presence of MMC enhances the stability of G‐quadruplex assemblies, even after additional covalent crosslinking.

Since the ThT fluorescence assay confirmed the formation of G‐quadruplex structures prior to TG‐mediated crosslinking, the same fluorescence probe was used to evaluate the influence of Ficoll and its concentration on the temporal stability of these supramolecular assemblies within the hybrid hydrogels, under biologically relevant conditions (Figure 2b). Analysis of the ThT fluorescence profile of the hybrid hydrogels with (12.5 and 25 mg/mL) and without Ficoll, measured before incubation in culture medium (day 0), revealed the same trend observed before TG‐mediated crosslinking, i.e., a decrease in the fluorescence intensity upon Ficoll incorporation (Figure 2c). Furthermore, the gelatin‐only control experiment, which lacks G‐quadruplex structures, exhibited no fluorescence increase, confirming the selectivity and reliability of the assay for monitoring G‐quadruplex content over time (Figure 2c; Figure S6a). At subsequent time‐points, ThT fluorescence was measured again (Figure S6b), and the G‐quadruplex disassembly was calculated as the percentage of fluorescence decrease relative to the initial value at day 0, which was set as 100%. As shown in Figure 2d, a notable difference was observed in the disassembly after 1 day.

While the Ficoll‐free hydrogels underwent 47% disassembly, the Ficoll‐containing hydrogels displayed substantially lower disassembly, with 26 and 8% for the lower and higher Ficoll concentrations, respectively. After 14 days, the Ficoll‐free hydrogels experienced a disassembly of 67%, while the hydrogels containing Ficoll showed reduced disassembly rates of 33% and 46%, depending on the concentration. Such differences in the disassembly rate can be correlated with the water content of the hydrogels, as water molecules play a crucial role in the stability of the G‐quadruplex supramolecular structures. To investigate this interdependence, the water content of the hydrogels with and without Ficoll was evaluated after 1 and 3 days of incubation in culture medium (Figure 2e). On day 1, the water content decreased as the Ficoll concentration increased. However, by day 3, no significant differences were observed between the different hydrogel formulations. While Ficoll — especially at higher concentrations — effectively reduced the hydrogel's water content, its absence led to full hydration by day 1, which in turn resulted in increased disassembly rates. These results highlight the importance of reducing the quantity of water at earlier stages, as it strongly enhances the long‐term stability of the supramolecular structures. Although Ficoll at 12.5 mg/mL did not significantly affect the water content when compared to the Ficoll‐free condition, it still enhanced the stability of the supramolecular assembly. This suggests that the G‐quadruplex stability is not solely determined by the quantity of water molecules but also by their activity around the supramolecular structure [34].

Ficoll is often assumed to behave as a hard sphere, resulting in excluded volume effects that reduce the available space to free water molecules (Figure 2f) [107, 108, 109]. Moreover, the presence of hydroxyl groups in Ficoll's chemical structure facilitates hydrogen bonding interactions between the polymer and the surrounding water, leading to the formation of a highly dense hydration shell that shields the G‐quadruplex structure from water [110, 111]. Since water molecules heavily interact with Ficoll's surface, their diffusion toward the G‐quadruplex structure is hindered, contributing to its increased stability. Such behavior is consistent with established theoretical models using Ficoll as a macromolecular crowding agent, based on excluded‐volume and hydration‐mediated mechanisms [112, 113, 114], as well as with molecular dynamics simulations that capture hydration dynamics under crowded conditions [111, 115]. Overall, these results suggest that the long‐term stability of the G‐quadruplex hydrogels is due to the synergistic effect of dehydration, promoted by the presence of Ficoll, and in‐bath covalent crosslinking, mediated by TG. Other approaches have succeeded to produce stable G‐quadruplex hydrogels using organic‐aqueous mixtures (e.g., ethanol:water) [116], ferrocene‐boronic acid derivatives that increase hydrogel hydrophobicity [31], or toxic metal cations (e.g., dimercury (I)) [117]. However, these methods are incompatible with cell encapsulation, either due to the presence of toxic metals and organic solvents, or by the increased hydrogel hydrophobicity, which impairs nutrient diffusion to the cells. In fact, achieving long‐term stability under physiological conditions, while maintaining appropriate biological performance, remains as a major challenge in the field. Recently, a polyethyleneimine coating strategy has been reported to extend the stability of G‐quadruplex hydrogels up to 7 days [51]. Nevertheless, hydrogel stability was assessed solely through scaffold area measurements, which do not fully capture underlying processes such as G‐quadruplex disassembly. Altogether, these works highlight the major challenge of attaining G‐quadruplex hydrogel stability and underscore the need for new strategies that not only support cell viability and enable long‐term stability, but also preserve the integrity of the G‐quadruplex supramolecular structure under biologically relevant conditions, an aspect often overlooked in the literature.

### 2.4 Rheological Characterization of the G‐Quadruplex Supramolecular Hydrogel Inks

#### 2.4.1 Effect of Macromolecular Crowding on Ink Gelation and Flow Properties

**FIGURE 3:**
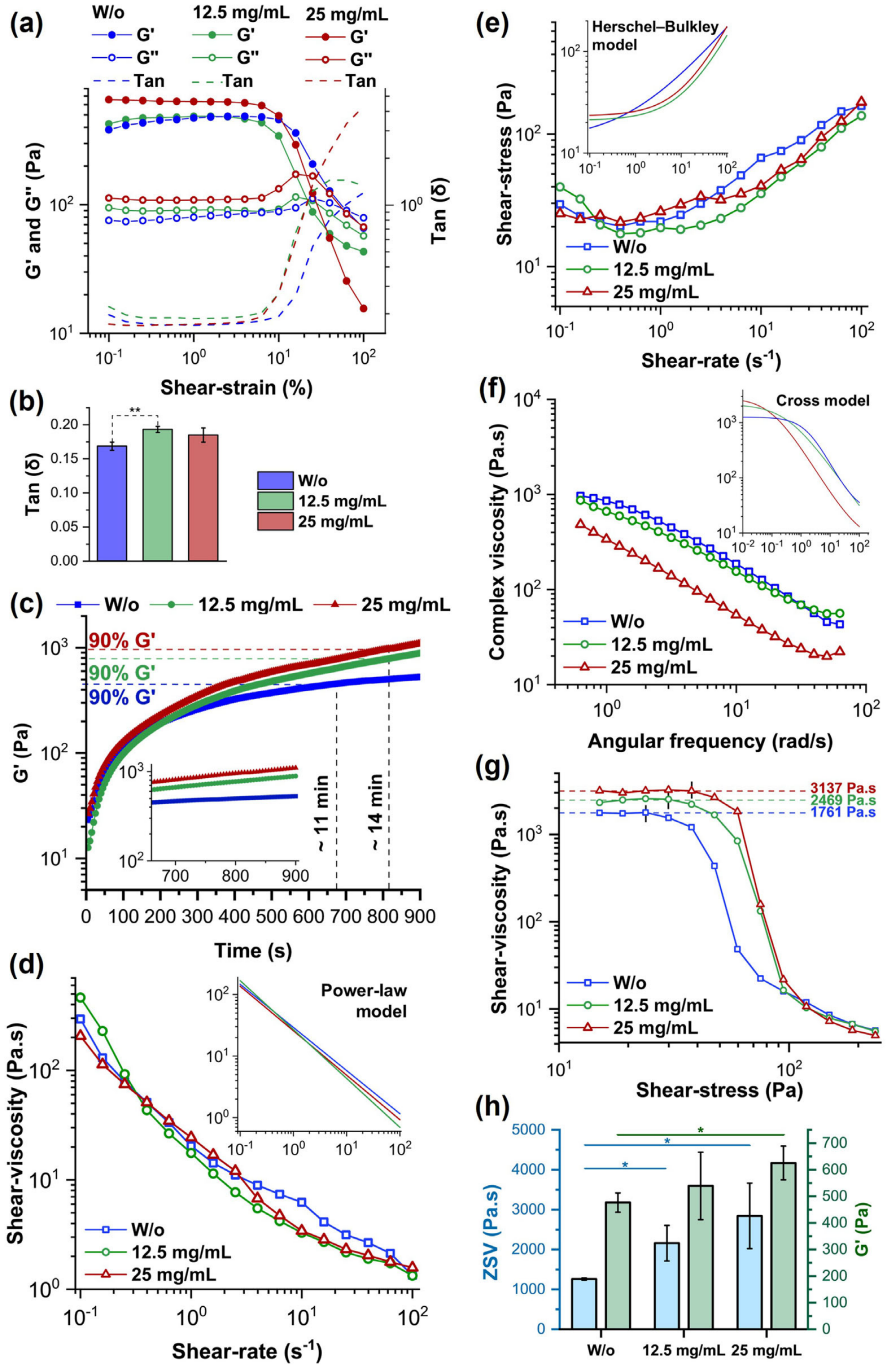
Rheological characterization of the gelatin‐based G‐quadruplex‐derived hydrogel inks: (a) strain sweep measurements of all tested inks to determine the LVR and the 𝛾_y_ (G′ = G′′); (b) tan δ determined as the mean of G′′/ G′ ratios within the LVR (*N* = 3); (c) time sweep tests performed for all formulations at 0.1% strain (within the LVR) and 1 Hz frequency; (d) shear‐viscosity as a function of shear‐rate fitted with the power‐law model; (e) Herschel‐Bulkley model applied to the shear‐stress vs. shear‐rate plot; (f) complex viscosity as a function of angular frequency fitted with the Cross model; (g) shear‐stress ramp, with colored dashed lines representing the zero‐shear‐viscosity (ZSV) of different formulations and a black line highlighting the yield stress; (h) comparison of stiffness and ZSV (*N* = 3). All the experiments were performed at 37°C; data represents the mean of three replicates. Statistical analysis was performed using an unpaired *t*‐test. Individual significance levels are indicated as follows: ^*^ *p* < 0.05, ^**^ *p* < 0.01. In (h), green and blue significance levels are relative to G′ and ZSV, respectively.

Oscillatory amplitude sweep assays were carried out to assess the viscoelastic properties of the inks, namely the linear viscoelastic region (LVR), yield strain (𝛾_y_), and the loss tangent (tan δ = viscous modulus (G′′) / elastic modulus (G′)). As shown in Figure 3a, all formulations exhibited an LVR region at lower shear‐strains, characterized by a strain‐independent viscoelastic behavior. The onset of non‐linear behavior marks the end of the LVR and the beginning of a gel‐sol phase transition. Although this behavior was observed in the inks both with and without Ficoll, the crossover point where G′ = G″ (𝛾_y_) [118] revealed to be lower for the inks containing MMC. As previously shown in Figure S5c, the absence of MMC leads to a less dense and more dispersed G‐quadruplex‐derived fiber network, providing more available space for fiber mobility without disrupting their interactions or entanglements under applied strain. We hypothesize that this increased freedom allowed the ink to sustain deformation over a broader strain range. In contrast, the presence of MMC induces a more densely packed fiber organization, thereby restricting the fiber rearrangement and thus reducing the 𝛾_y_. A similar behavior has been previously reported in PEG‐based microfibers, where increased available space and interstitial fluid content lead to higher 𝛾_y_ values [119]. Another parameter that should not be overlooked in the amplitude sweep experiments is the damping factor, also known as tan δ (Figure 3b). Although an increase in tan δ was observed for both formulations containing Ficoll, this increase was only significant for the one with the lowest MMC concentration. Since higher tan δ values have been correlated with better filament uniformity [120], these findings suggest a potentially improved filament printability of the Ficoll‐containing inks, particularly at 12.5 mg/mL.

To further assess the influence of macromolecular crowding environments on the gelation kinetics, G′ was monitored over time in all formulations (Figure 3c). To enable a meaningful comparison across formulations, we defined the gelation time as the time required to reach 90% of the final G′ value, a threshold that allows a consistent evaluation of the gelation time even when a well‐defined plateau is not observed. Our results reveal that in the absence of Ficoll, the ink displayed a well‐defined G′ plateau, while the inks containing Ficoll (12.5 and 25 mg/mL) showed a clear tendency toward stabilization but did not reach a full G′ plateau within the experimental timeframe (Figure 3c, inset). This behavior reveals that the presence of the MMC delayed the gelation process, with 90% of G′ being reached after ∼14 min for both Ficoll concentrations — about 3 min later than in the Ficoll‐free condition (∼11 min). These findings suggest that macromolecular crowding delays the assembly process [121, 122, 123], resulting in an increased gelation time.

Having already demonstrated the influence of the MMC on gelation kinetics, we subsequently investigated its effect on the viscosity profiles. Classical rheological parameters such as shear‐thinning, yield stress, and zero‐shear‐viscosity are well‐established as predictors of ink printability, which are intrinsically linked to the underlying molecular architecture [46, 124, 125]. Therefore, rheological measurements were conducted to evaluate the shear‐thinning behavior of the inks with and without Ficoll. For all ink formulations, a decrease in the viscosity was observed while increasing the shear‐rate, which is characteristic of a non‐Newtonian shear‐thinning behavior (Figure 3d). By fitting the data to a power‐law model (parameter details provided in Table S1), flow index (*n*) values were obtained, with all inks exhibiting *n* values within the shear‐thinning range of 0.206 ≤ *n* ≤ 0.299. Among them, the ink containing 12.5 mg/mL of Ficoll exhibits the lowest *n* value, demonstrating a more pronounced shear‐thinning behavior, which could potentially lead to a higher printing fidelity (Table 1).

**TABLE 1:** Viscosity profiles of gelatin‐based G‐quadruplex‐derived hydrogel inks with different concentrations of Ficoll: the flow index was obtained using the power law model and subsequently used to calculate the shear‐rate at the wall (𝛾̇_wall_). The inks’ yield stress (𝜎_0_) and zero‐shear viscosity (η_0_) were predicted by Herschel‐Bulkley and Cross models, respectively.

Gelatin‐based G‐quadruplex‐derived inks	Printing parameters		Power law model			Herschel–Bulkley model		Cross model	
	Needle diameter (mm)	Printing speed (mm/s)	n	𝛾̇_wall_ (s^−1^)	R^2^	𝜎_0_ (Pa)	R^2^	η_0_ (Pa.s, ZSV)	R^2^
**W/o Ficoll**	0.410	8	0.299	**247.590**	0.968	13.48	0.983	1260	0.999
		9		278.538					
		10		309.487					
		12		371.384					
		14		**433.282**					
**W/Ficoll_12.5 mg/mL_**	0.410	8	0.206	**306.512**	0.925	20.96	0.962	2161	0.999
		9		344.826					
		10		383.140					
		12		459.768					
		14		**536.396**					
**W/Ficoll_25 mg/mL_**	0.410	8	0.278	**257.449**	0.972	23.20	0.997	2842	0.999
		9		289.630					
		10		321.811					
		12		386.173					
		14		**450.535**					

These *n* values were then employed to estimate the shear‐rates experienced during extrusion, a critical parameter in 3D bioprinting, as high shear‐rates can potentially lead to cell damage [126, 127]. Assuming five different printing speeds (8, 9, 10, 12, and 14 mm/s) and a fixed nozzle diameter (22G, 410 µm), the shear‐rates at the wall (𝛾̇_wall_) were calculated using Equation S1 [46]. Across all tested printing speeds, the highest 𝛾̇_wall_ values were observed for the formulation containing 12.5 mg/mL of Ficoll, which exhibited the lowest *n* value (Table 1). It is also noteworthy that, although excessive shear‐rates can compromise cell viability, a certain shear‐rate threshold is required to induce sufficient viscosity drop, adequate flow behavior, and smooth filament formation [125]. Although the power‐law model provides valuable insights into the shear‐thinning behavior, it fails to account for yield stress. To capture both the initial resistance to flow and the subsequent shear‐dependent response, we further employed the Herschel–Bulkley empirical model (parameter details provided in Table S1). As expected, the formulated inks do not spontaneously flow in the absence of applied shear forces, indicating the presence of a yield stress (𝜎_0_), i.e., a minimum shear‐stress must be exceeded to initiate flow [46, 128]. As showcased in Figure 3e and Table 1, the incorporation of MMC leads to an increase in 𝜎_0_, ranging from 13.48 Pa for the Ficoll‐free ink to 23.20 Pa for the ink containing 25 mg/mL of Ficoll. These values corroborate those previously reported for other supramolecular inks [129, 130].

In cellular microenvironments, macromolecular crowding limits the fluid mobility and enhances the viscosity [131]. To assess if the incorporation of the MMC within the G‐quadruplex supramolecular system leads to a similar phenomenon, a Cross model (parameters details provided in Table S1) was employed to fit the complex viscosity as a function of the angular frequency (Figure 3f and Table 1). This model enabled the prediction of the zero‐shear‐viscosity (ZSV, η_0_), a key parameter that reflects resistance to flow under near‐static conditions [132]. Our results indicate that the ZSV also increases while increasing the MMC concentration, which was expected given the previously predicted values for 𝜎_0_. In addition to model‐based predictions, shear‐stress ramp assays can also be used to determine the yield stress, the associated viscosity drop, and the viscosity at rest (Figure 3g; Table S2). A closer examination of the shear‐stress ramp data reveals that all formulated inks exhibit an initial resistance to flow, as shown by the initial viscosity plateau at low shear‐stress values, which can be taken as the viscosity at rest or ZSV. However, after applying a shear‐stress that surpasses the yield stress — here defined as the point after which viscosity begins to decrease — the inks start to flow. Once again, the presence of MMC increases both the η_0_ and 𝜎_0_, in agreement with the behavior predicted by the empirical model. Although slightly higher values were obtained from the shear‐stress ramp assay, the tendencies for each type of ink were maintained (Table S2). Another interesting observation was the clear association between higher 𝜎_0_ and greater viscosity drop with increasing Ficoll concentration. In fact, such a combination of high yield stress and large viscosity drop has been proposed as a fundamental requirement for filament formation [124].

Given that the printing fidelity can be influenced by both the ink stiffness and viscosity, the ability to independently modulate these two variables is paramount for elucidating their specific effect in 3D bioprinting [133]. In this context, the aforementioned ZSV values (Table 1) were plotted against the ink's stiffness, taken as the G′ values at 1 Hz [9, 134] (Figure 3h). The gathered results showcased that lower concentrations of Ficoll induce a significant increase in the viscosity, while exerting negligible influence on stiffness. On the other hand, higher concentrations of Ficoll significantly increase both parameters, suggesting that the decoupling of stiffness and viscosity at rest occurs within a certain range of MMC concentrations.

#### 2.4.2 Thixotropic and Self‐Healing Behavior

**FIGURE 4:**
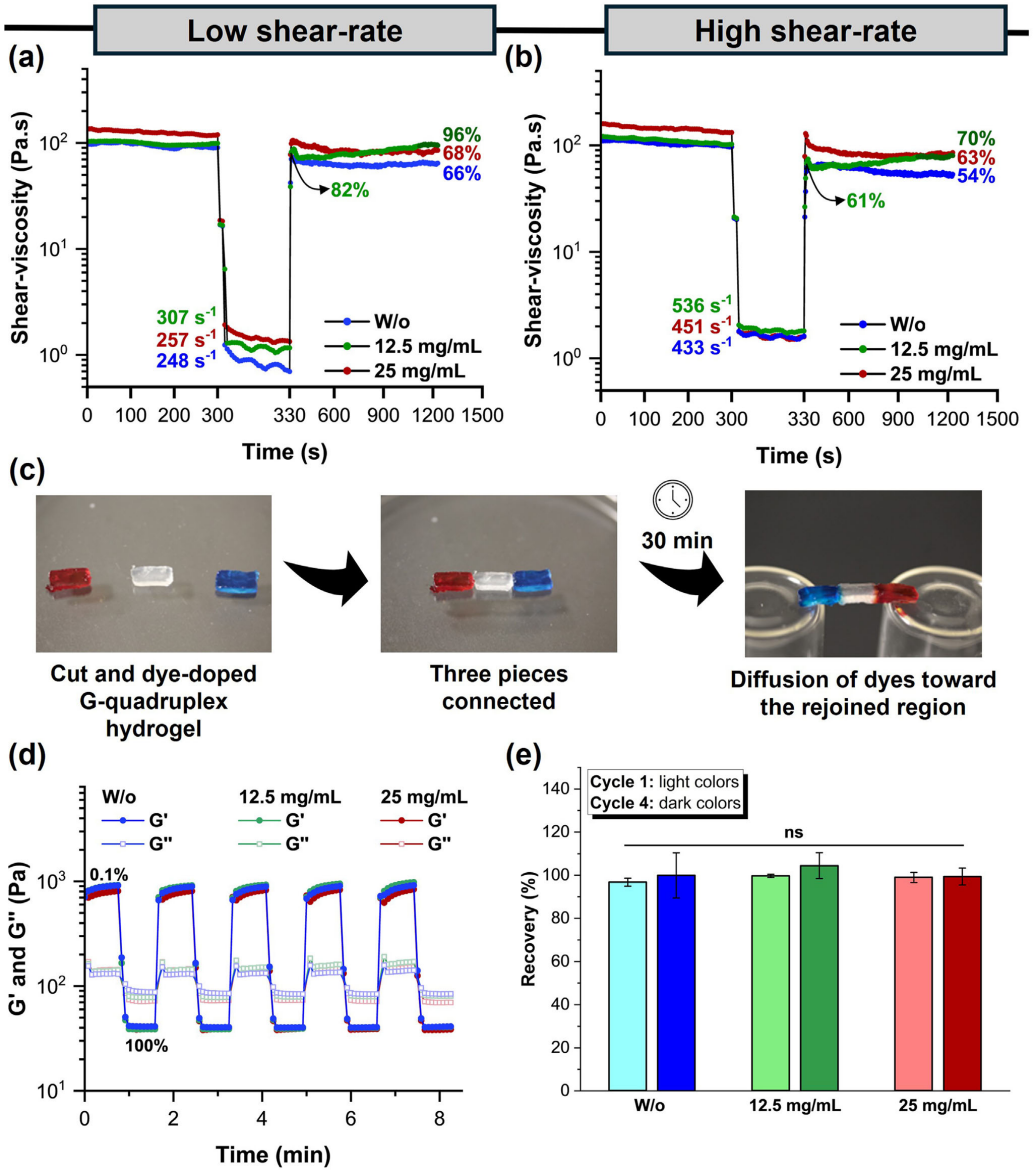
Thixotropic behavior and self‐healing ability of the gelatin‐based G‐quadruplex‐derived hydrogel inks: thixotropic behavior of all tested inks under low (a) and higher shear‐rates (b). Shear‐rates and recovery percentages, represented by red and blue, correspond to formulations with 25 mg/mL, and no Ficoll, respectively, while green corresponds to the formulation with 12.5 mg/mL of Ficoll, with dark green indicating the recovery percentage after reaching the plateau; (c) digital images depicting the self‐healing behavior of the MMC‐containing G‐quadruplex hydrogel; (d) step‐strain sweep assay under alternating low (0.1%) and high (100%) shear‐strains for all tested inks; (e) recovery percentages of all formulated inks after 1 and 4 deformation cycles (*N* = 3). Statistical analysis was performed using an unpaired t‐test, with differences considered significant at *p* < 0.05.

Thixotropy is a time‐dependent behavior that ensures lower viscosity during extrusion — due to the increasing shear‐rates at the nozzle wall — allowing the ink to recover its viscosity after printing, a crucial parameter for preventing filament spreading and maintaining its structural integrity [135]. In this assay, the previously calculated 𝛾̇_wall_ values for both the highest (14 mm/s) and lowest (8 mm/s) printing speeds (Table 1) were used to assess the thixotropic behavior of all tested inks, aiming to evaluate viscosity recovery under shear conditions that mimic real extrusion scenarios. To facilitate the analysis, two shear‐rate groups were defined for all inks: a lower shear‐rate and a higher shear‐rate group, corresponding to 𝛾̇_wall_ at 8 and 14 mm/s, respectively. Within the lower shear‐rate group, the ink containing 12.5 mg/mL of Ficoll displayed the most pronounced thixotropic behavior, despite being subjected to a superior shear‐rate than the other samples. This was evidenced by the highest initial recovery percentage of 82%, which increased to 96% upon reaching a plateau after 13 min (Figure 4a). However, this behavior was not observed for both the ink with 25 mg/mL of Ficoll and the Ficoll‐free ink, which exhibited a plateau throughout the entire recovery step, with lower recovery percentages of 68% and 66%, respectively. Conversely, although in the higher shear‐rate group viscosity recovery was compromised for all inks, their recovery tendency was maintained, with the 12.5 mg/mL of Ficoll exhibiting the highest recovery percentage (70%) and the Ficoll‐free ink the lowest (54%) (Figure 4b). Additionally, differences in the recovery percentages between Ficoll concentrations were less evident at high shear‐rates, which resulted from the substantial loss in viscosity recovery of the 12.5 mg/mL Ficoll ink when compared to low shear‐rates. Overall, the presence of Ficoll promoted more pronounced thixotropic behavior with enhanced recovery percentages. This effect was more evident when lower shear‐rates were applied, with Ficoll concentration having a stronger influence. Moreover, these findings suggest a reduced microstructural recovery following extrusion at high printing speeds, which corroborates previous reports on conventional extrusion‐based 3D printing [136].

While the combination of shear‐thinning and yield stress behavior allows for smooth extrusion through the nozzle and the maintenance of the filament shape after deposition without resorting to high printing pressures, it is also important to evaluate the self‐healing behavior of the ink. The latter would promote cohesive merging between the deposited layers and contribute to the overall structural integrity of the printed construct [128, 137]. In this context, the self‐healing ability of the formulated inks was qualitatively assessed by cutting the hydrogel into three pieces, staining two of them with different dyes, and promoting their contact for ca. 30 min at 37°C without any external stimulus. As shown in Figure 4c, the ink is capable of fully healing into a single, integral piece as demonstrated by the diffusion of the dyes toward the rejoined region, which remains cohesive even when suspended. In addition, alternate step‐strain sweep tests were conducted aiming to evaluate the autonomous recovery of the inks to their original viscoelastic properties after four deformation cycles (Figure 4d). As expected, under lower strains (0.1%), the inks retained their gel‐like characteristics (G′ > G′′), whereas at higher strains (100%), the inverse was verified (G′′ > G′), indicating that the hydrogels underwent a transient gel–sol phase transition. When the lower strains were reapplied, all inks demonstrated a rapid and complete recovery of their original G′ values, with recovery values of approximately 97%, 100%, and 99% for the inks without and with 12.5 or 25 mg/mL of Ficoll, respectively (Figure 4e). This remarkable self‐healing ability showed no significant changes even after four deformation cycles, arising from the dynamic covalent bonds and supramolecular nature of the developed inks, which allows the re‐establishment of crosslinks and the restoration of their original microstructure. Although the incorporation of Ficoll into the 3D network resulted in different thixotropic profiles, particularly at 12.5 mg/mL, it did not influence the inherent self‐healing behavior of the inks. Based on the gathered data, we hypothesize that, under applied shear, the inclusion of the MMC has a more pronounced effect on the recovery of viscosity rather than in the elasticity [138].

### 2.5 Screening the Filament, Mesh, and Shape‐Fidelity of the G‐Quadruplex Hydrogel Inks

**FIGURE 5:**
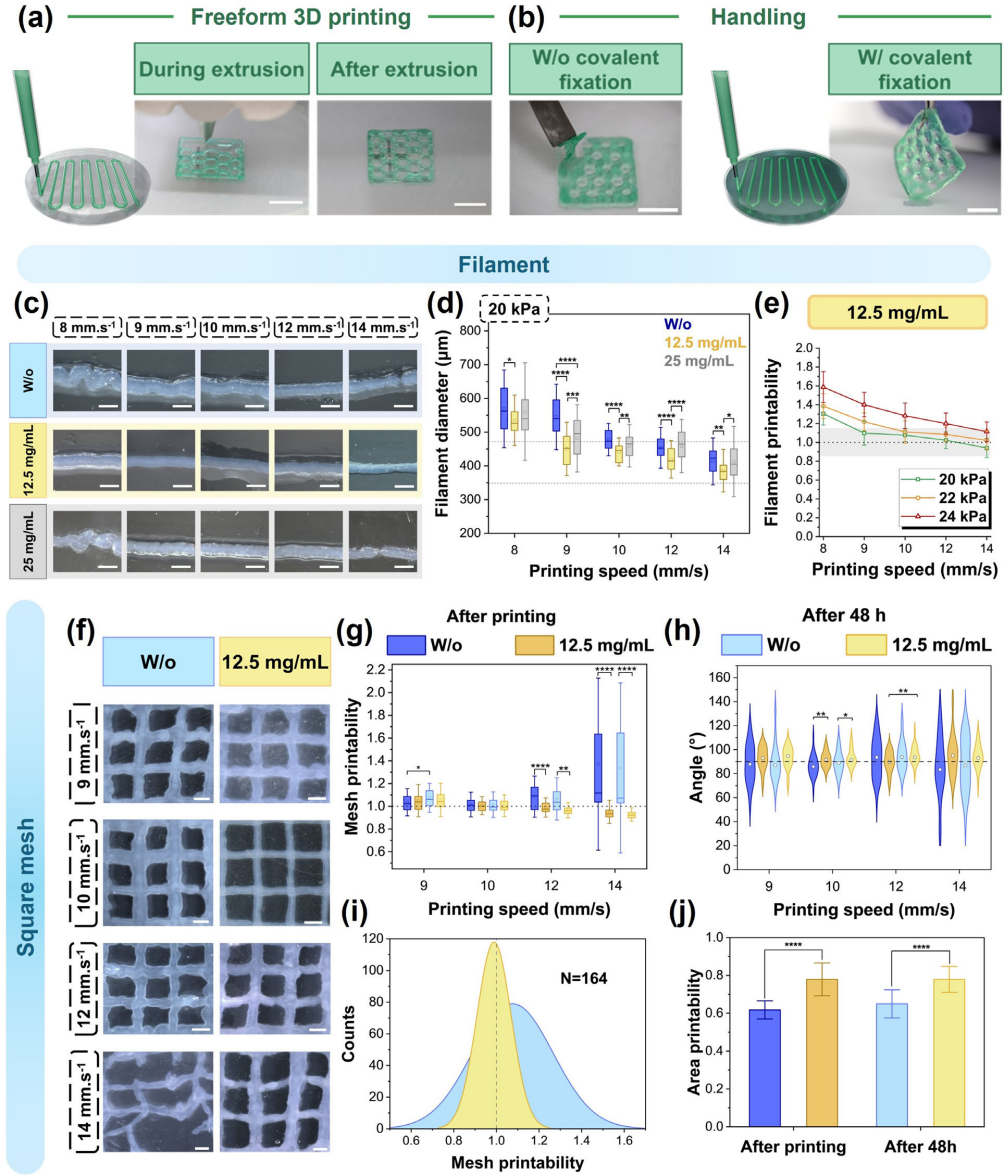
Toward the optimization of the printing settings: (a) freeform printing of the 12.5 mg/mL Ficoll ink; (b) digital images showing grid handleability before and after covalent reinforcement; (c) stereomicroscopic images and (d) filament diameter evaluation of the printed filaments at a constant printing pressure of 20 kPa (*N* ≥ 50 measurements per speed obtained from at least six printed filaments); (e) filament printability of the 12.5 mg/mL Ficoll ink at different printing pressures as a function of printing speed, with the acceptable printability region highlighted in grey (for 20 kPa, *N* ≥ 50; for 22 kPa, *N* ≥ 39; for 24 kPa, *N* ≥ 39 measurements per speed obtained from at least six printed filaments); (f) stereomicroscopic images of the printed grids; (g) mesh printability (*N* = 45 measurements per speed obtained from at least three printed square mesh grids, except for the Ficoll‐free formulation at 14 mm/s, where some of the mesh grids were fragmented and their printability could not be assessed) and (h) internal mesh angle (*N* = 36 measurements per speed obtained from at least three printed square mesh grids) determination of the square mesh constructs, evaluated both after printing (dark colors) — following extrusion and covalent fixation — and after 48 h of incubation in culture medium at 37°C (light colors); (i) effect of Ficoll on the distribution of mesh printability values after 48 h of incubation in culture medium (*N* = 164); (j) area printability (*N* = 9). Scale bars: 1 cm in (a), 1 mm in (c) and (f). Statistical analysis was performed using a Kruskal‐Wallis test followed by Dunn's post hoc test for (d), a Mann‐Whitney *U* test for (g) and (h), and an unpaired t‐test for (j). Individual significance levels are indicated as follows: ^*^ *p* < 0.05, ^**^ *p* < 0.01, ^***^ *p* < 0.001, and ^****^ *p* < 0.0001.

Interestingly, owing to its favorable rheological properties, the MMC‐containing supramolecular ink can be printed in the absence of a supporting bath, without apparent filament spreading (Figure 5a), highlighting its potential for freeform 3D printing. However, similarly to other supramolecular systems [58, 139, 140, 141], its poor mechanical strength poses a major hurdle for the successful detachment and handling of the final 3D printed construct (Figure 5b, left). To overcome these limitations, supporting baths offer a versatile strategy by providing physical confinement during printing while simultaneously serving as reservoirs for secondary crosslinking, thereby enabling the successful fabrication of soft materials into complex 3D structures [142, 143, 144, 145]. In this context, an agarose bath supplemented with 5% (w/v) of TG was employed, enabling in situ enzymatic covalent crosslinking while providing physical support for the fabrication of gravity‐defying constructs. The rheological properties of the supporting bath were then evaluated, confirming that the shear‐thinning behavior is preserved even after TG incorporation, allowing for a smooth printing process (Figure S7a). Furthermore, the TG‐containing supporting bath exhibited thixotropic behavior, restoring its original viscosity upon removal of the shear‐rate (Figure S7b). Altogether, these findings highlight the bath's capacity to endure and recover from the mechanical perturbations imposed throughout the printing process.

Having established that TG incorporation does not compromise the rheological properties of the supporting bath, the inks were printed into the TG‐containing agarose bath (see Video S1), with both the printhead and bath maintained at 37°C, a temperature above the upper critical solution temperature of gelatin to prevent its temperature‐induced self‐gelation [146]. Afterward, the printed constructs were incubated for 2 h at 37°C and washed prior to characterization. In contrast to its counterpart, these hybrid constructs — formed through a combination of supramolecular and covalent crosslinking — could be easily handled, exhibiting improved mechanical integrity (Figure 5b, right) and being able to withstand substantial deformation without permanent damage (see Video S2). After demonstrating the success of the implemented hybrid approach in ensuring construct integrity, the filament fidelity was evaluated by measuring the diameter of the extruded filaments and comparing it to the theoretical inner nozzle diameter (22G, 410 µm), laying the groundwork for subsequent analysis of more complex factors such as shape‐fidelity and structural complexity. Filament uniformity was evaluated using three printing pressures (20, 22, and 24 kPa) and five printing speeds (8, 9, 10, 12, and 14 mm/s). Qualitative data are presented in Figure 5c and Figure S8a,b, and quantitative results are presented in Figure 5d and Figure S8c,d. As expected, increasing the printing speed at constant pressure resulted in thinner filaments across all formulations, with some filament breaks observed at the highest printing speed (14 mm/s) and lowest printing pressure (20 kPa). On the other hand, the increase in printing pressure resulted in the formation of less uniform (Figure S8a,b) and thicker filaments (Figure S8c,d). Notably, a closer examination of the box plots reveals that differences in the filament diameter between inks become not statistically significant as the pressure increases (Figure 5d; Figure S8c,d). In fact, the data showcased that a printing pressure of 20 kPa provides a broader range of printing speeds within the acceptable printability region for all inks, making it the optimal printing pressure for further 3D printing assays (Figure 5e; Figure S8e,f).

Moreover, as predicted by the rheological data, the presence of the MMC improved the filament fidelity. However, this behavior happened in a concentration‐dependent manner, with the lowest concentration of Ficoll producing more uniform and homogeneous filaments (Figure 5c) within the optimal diameter range (Figure 5d). Therefore, this ink formulation was selected for further studies. In fact, at a printing speed of 10 mm/s, this ink formulation exhibited a deviation from the ideal filament diameter of less than 10% (filament diameter 441.4 µm; nozzle diameter 410 µm), indicating a significant improvement over the ∼22% deviation (filament diameter 337.8 µm; nozzle diameter 435 µm) reported at the same printing speed for a gelatin‐functionalized bioink based on complementary aptamer hybridization [147].

After optimizing the printing pressure and identifying the optimal Ficoll‐based ink formulation, the mesh printability (printability = 1 indicating a perfect square) and the angle distribution of the square mesh constructs (L × W; 20 mm × 20 mm) were evaluated [48]. Figure 5f shows magnified optical images of the printed square mesh grids at a constant pressure of 20 kPa with varying printing speeds, except for 8 mm/s, which, in the previous analysis, consistently resulted in thicker filaments with the lowest fidelity. The presence of Ficoll clearly enhanced the mesh printability, producing well‐defined square patterns with printability values (*Pr)* closest to 1, particularly at a printing speed of 10 mm/s (Figure 5f,g). In contrast, the MMC‐free ink exhibited noticeable over‐deposition and non‐uniform filaments, with some evidence of fragmentation, leading to distortion of the mesh geometry, especially at 12 and 14 mm/s (Figure 5f). This behavior translated into a reduced mesh printability, broader distribution in the corresponding box plots, and relatively large error bars for these data points (Figure 5g). In fact, both in the presence or absence of the MMC, the highest printing speed resulted in a reduced mesh printability, which could be related to the diminished viscosity recovery after extrusion, as anticipated by the rheology data (Figure 4b). While the printability factor offers a measure of how closely the printed structures resemble perfect squares, it does not account for deviations in internal angles [148]. To address this limitation, the internal mesh angle distribution was also evaluated, showing that a printing speed of 10 mm/s yielded a more homogenous mesh angle distribution of about 90°, as evidenced by the reduced distribution of values (Figure 5h). In contrast, and consistent with the mesh printability evaluation, the highest printing speed resulted in a more heterogeneous angle distribution, being a consequence of mesh distortion and fragmentation. Notably, the MMC‐containing ink exhibited an angle deviation of approximately 2%, representing a marked improvement when compared to the 16% deviation previously reported for G‐quadruplex‐based inks [51]. Knowing that mesh angles of about 90° and *Pr* close to 1 indicate well‐defined square grids with optimal perpendicular filament intersection, these results reveal that a printing speed of 10 mm/s ensures consistent pore dimensions with minimal structural defects [148]. In addition, to assess the structural stability of the 3D printed constructs, these parameters were re‐evaluated after 2 days of incubation in culture medium at 37°C. In most cases, no significant changes were observed in both parameters over time, indicating that the printed grids remained intact under physiological conditions. These results underscore the excellent stability of the constructs, which preserve their geometrical fidelity and printability even after 48 h in a biologically relevant environment. To enable a direct evaluation of Ficoll's effect on printability, irrespective of the printing speed, all mesh *Pr* obtained at 20 kPa were pooled and their distributions analyzed (Figure 5i). The resulting distribution curves reveal that the Ficoll‐containing ink exhibits a more narrowly distributed curve centered around a *Pr* of 1. This indicates that, regardless of the printing speed, the ink containing Ficoll exhibits higher printability at 20 kPa, excelling its counterpart lacking Ficoll. A similar trend has been reported for methacrylated gelatin‐based inks, in which the incorporation of methylcellulose enhanced printability, leading to higher *Pr* values [149].

Lastly, the area printability (Figure 5j) and pore area (%) (Figure S8g) of both ink formulations were evaluated. The data revealed that both parameters remained unaffected after 48 h of incubation under physiological conditions, further emphasizing the potential of the developed hybrid G‐quadruplex‐derived inks for high‐fidelity 3D bioprinting. Notably, this performance contrasts with previously reported G‐quadruplex inks, which exhibited a 25% reduction in pore area within the first 24 h of incubation in culture medium [51]. Furthermore, the obtained pore area values were within the optimal range of 40%–70%, recommended for tissue engineering applications [150]. While MMC incorporation did not affect pore area, it significantly improved area printability. Nevertheless, in both inks, this value remained below 0.8, suggesting limitations in achieving an ideal area fidelity.

### 2.6 Unveiling the Role of In‐Bath TG‐Mediated Covalent Fixation

**FIGURE 6:**
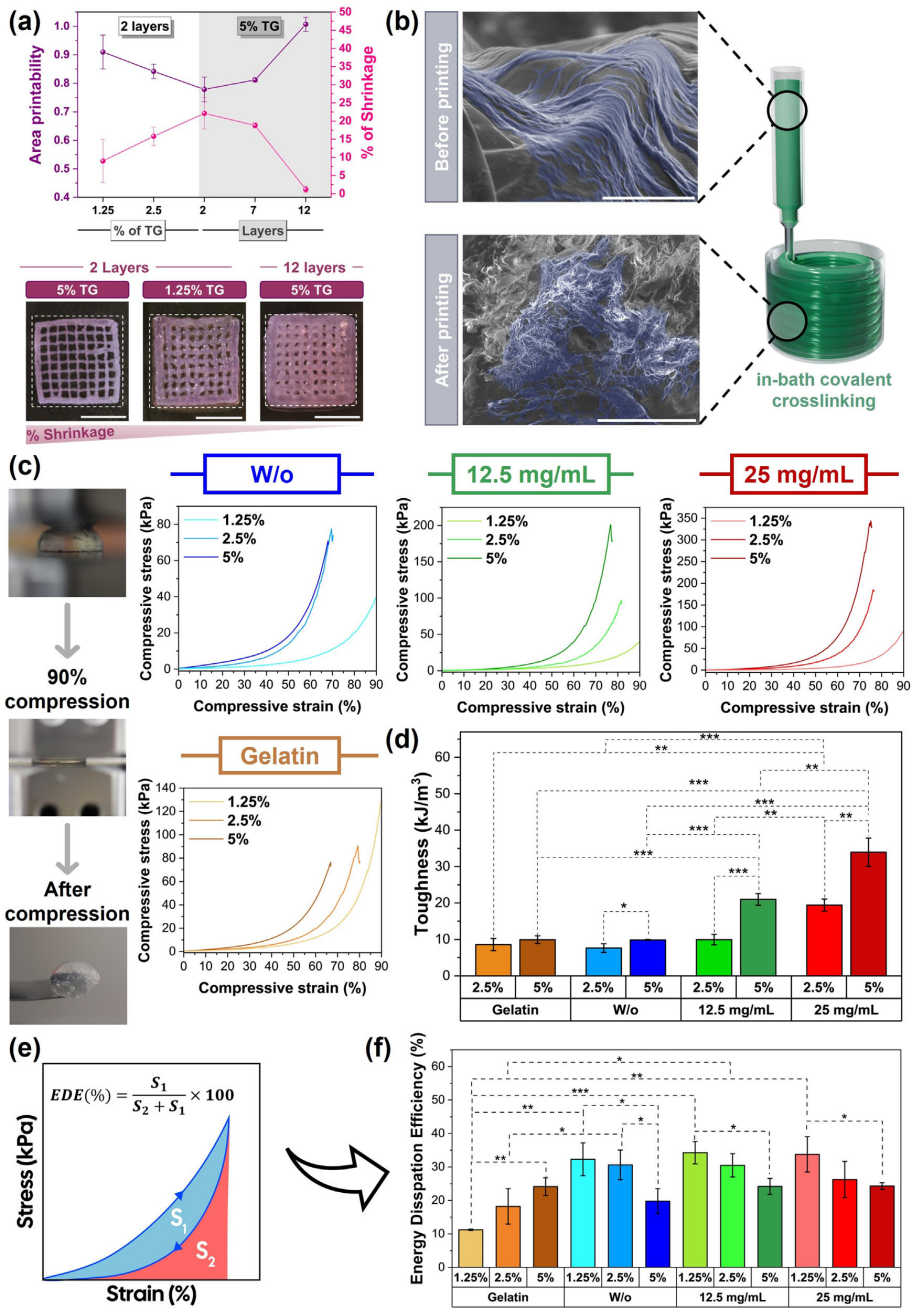
Unveiling the role of in‐bath TG‐mediated covalent fixation: (a) shrinkage and area printability evaluation (*N* = 9) as a function of the TG concentration and the number of printed layers (top), and digital images of the printed grids (bottom). Scale bars: 1 cm; (b) SEM images of the ink pre‐ and post‐printing (after extrusion and in‐bath covalent fixation using 5% (w/v) of TG), with fibrous topology highlighted in blue. Scale bars: 50 µm; (c) unidirectional compressive strain/stress curves and digital images of the hybrid hydrogel after being compressed to 90% strain (left); (d) toughness evaluation (*N* = 3); (e) schematic representation of the method used to calculate energy dissipation efficiency (EDE), adapted with permission from ref [162]. Copyright 2022 Wiley‐VCH GmbH; (f) EDE assessment (*N* = 3). Statistical analysis was performed using an unpaired t‐test. Individual significance levels are indicated as follows: ^*^ *p* < 0.05, ^**^ *p* < 0.01, and ^***^ *p* < 0.001.

The previously ascertained deviation from ideal area fidelity prompted us to investigate the construct's shrinkage as a function of the TG concentration (Figure 6a, top). The degree of shrinkage was found to be tunable by varying the TG concentration, with higher concentrations leading to increased shrinkage and reduced area printability, as expected. This effect was attributed to a greater density of crosslinking points arising from higher TG concentrations [151], resulting in a more tightly crosslinked network. Consequently, macroscopic contraction of the printed grids occurred, which could be minimized by using a lower TG concentration (1.25% (w/v)) (Figure 6a). However, this came at the cost of compromised mesh fidelity (Figure 6a, bottom) and mechanical fragility, resulting in grids that, in contrast to the ones crosslinked with 5% (w/v) of TG, were difficult to handle. Additionally, we observed that, at a fixed TG concentration of 5% (w/v), increasing the number of the printed layers to 12 resulted in the lowest shrinkage (1.1%) and accurate values for area printability (*Pr* ∼ 1) (Figure 6a, top). This can be explained by the higher amount of deposited material, which forms a more robust construct that is less prone to TG‐induced shrinkage [152]. Remarkably, this paves the way for the fabrication of much more complex, multilayered structures where shrinkage can be mitigated, turning structural complexity into an effective strategy for preserving volumetric integrity. Since macroscopic shrinkage is inherently linked to changes in the underlying network architecture, we investigated the topology of the MMC‐containing G‐quadruplex ink pre‐ and post‐TG crosslinking (i.e., after printing) using scanning electron microscopy (SEM). All processing steps, including imaging, were carried out at 37°C to preserve native‐like structural features. As shown in Figure 6b, prior to extrusion, the supramolecular ink exhibits a well‐distributed network of elongated fibers. However, after the 3D printing process, which involves extrusion and subsequent covalent fixation, a topology shift from a loosely packed into a more compact and highly entangled fibrillar network was found. This network tightening correlates with the previously observed shrinkage, suggesting that the enzymatic crosslinking promotes network contraction. It is worth noting that, despite the morphological changes observed after covalent reinforcement, the material retains its fibrous topology, thus preserving its functionality as an ECM‐mimicking platform [58].

To further investigate the effect of TG‐mediated crosslinking on the viscoelastic behavior of the G‐quadruplex‐derived hydrogels, frequency sweep tests were conducted before and after covalent crosslinking (Figure S9a). Before TG‐mediated crosslinking, all formulations exhibited a gel‐like behavior, as revealed by a dominant elastic response (G′ > G′′) across the entire frequency range.

However, at high frequencies, it was possible to observe that the G′ increased, exceeding the values observed at lower frequencies. This behavior is characteristic of viscoelastic materials that dynamically adapt their elastic response (i.e., stiffness) under increasing frequencies [153, 154]. In particular, the hydrogel containing the lowest Ficoll concentration exhibited a more pronounced frequency‐dependent behavior, suggesting that the viscoelastic response is influenced by the MMC's concentration. After covalent crosslinking, an overall increase in G′ was observed for all formulations, as well as a greater difference between both moduli, revealing a stronger‐gel like behavior [155], which is consistent with the improved mechanical integrity of the 3D‐printed constructs following covalent reinforcement. Additionally, although both G′ and G′′ behaved similarly in the hybrid G‐quadruplex hydrogels, indicating that covalent fixation largely homogenized the viscoelastic behavior across all formulations, the gelatin control exhibited the greatest reduction in frequency dependence when compared to its non‐crosslinked counterpart (before covalent crosslinking) (Figure S9b). Its strong frequency‐dependent behavior prior to covalent crosslinking may induce flow without yield stress during 3D printing, an issue that in situ covalent fixation cannot mitigate, ultimately resulting in poor shape‐fidelity and inadequate interlayer cohesion, turning a hollow 3D model into a printed form with negligible porosity (Figure S9c).

To facilitate the analysis, the abovementioned G′ values for the supramolecular inks were plotted, this time directly compared with their hybrid counterparts, providing valuable insights into the effect of TG‐mediated crosslinking on stiffness. Figure S9d shows that, upon covalent crosslinking, a significant increase in stiffness was observed across all formulations, with 25 mg/mL of Ficoll yielding the highest G′ value (6.3 kPa). Interestingly, the greatest fold increase in stiffness (∼85 fold) occurs in gelatin alone. This can be attributed to the full availability of lysine ε‐amino groups for TG‐mediated crosslinking in the protein backbone, whereas in the gelatin‐based G‐quadruplex‐derived networks these groups are partially screened by the formation of imino‐boronate bonds with 2‐FPBA.

Following the rheological characterization, compression mechanical tests were carried out to further investigate the hybrid hydrogel's behavior under large deformations. Stress/strain curves were obtained for all conditions in the presence of different percentages of TG (1.25, 2.5, and 5%) (Figure 6c), from which Young's modulus, ultimate stress, and strain as well as toughness values were extrapolated. The Young's modulus of all formulations increased while increasing TG concentration (Figure S10a). Overall, hybrid hydrogels assembled in the absence of MMC (W/o), exhibited reduced stiffness across all TG concentrations when compared to fully covalent gelatin hydrogels, a trend also observed in ureidopyrimidinone‐based covalent/non‐covalent hybrid networks [156]. However, this difference diminished as the content of MMC increased, with the highest MMC concentration yielding Young's modulus comparable to gelatin control across all tested covalent crosslinking conditions. Notably, no significant differences were observed in stiffness between the 12.5 mg/mL Ficoll formulation and its Ficoll‐free counterpart, indicating that moderate MMC incorporation does not measurably influence network rigidity. Importantly, the hybrid hydrogels span a broad stiffness range, from softer environments at the lowest TG concentration under non‐crowded conditions (∼2 kPa) to markedly stiffer environments at the highest TG concentration under highly crowded conditions (∼12 kPa). This wide tunability further underscores the versatility of the developed system in matching the mechanical requirements of various human tissues, including brain (1–3 kPa) [157], adipose tissue (1.6–5.5 kPa) [157], and skeletal muscle (∼12 kPa) [158].

Interestingly, at 1.25% (w/v) of TG, the hydrogels did not break up to 90% strain, as confirmed both visually (Figure 6c, left) and by the strain‐stress curves. Since toughness represents the energy accumulated by the material up to failure, this parameter, along with ultimate stress and strain, were not evaluated for this TG concentration. As shown in Figure S10b, in the gelatin control, increasing TG concentration resulted in a significant decrease in ultimate strain. However, in the hybrid hydrogels, this parameter remains nearly constant across both TG concentrations. At 5% of TG, the hybrid hydrogels exhibit a significantly higher ultimate strain when compared to the gelatin control, a behavior not observed at 2.5% TG. Regarding ultimate stress measurements, the MMC‐containing hybrid hydrogels exhibited a reduction in ultimate stress when a lower TG concentration was used (Figure S10c). Despite this, the incorporation of the MMC resulted in significantly higher values, with the highest MMC concentration resulting in the highest ultimate stress across both TG concentrations. This increase in ultimate stress, facilitated by the spatial confinement (i.e., closer packing) of supramolecular fibers induced by MMC, also contributed to the higher toughness, which followed the exact same tendency as for the ultimate stress values (Figure 6d). A similar tendency has been observed in polyacrylamide hydrogels, where the addition of CaCl_2_ increased the spatial confinement and reduced the distance between the polymer chains [159], resulting in enhanced toughness as the CaCl_2_ concentration increased. Using the lower TG concentration, the differences in toughness were less pronounced, and only the formulation containing the highest Ficoll concentration exhibited a significant increase relative to gelatin control.

In summary, the gathered results suggest that for all tested formulations, varying the TG concentration exerted an evident influence on stiffness, while playing a minor role in dictating the endured strain. In addition, regardless of the TG concentration, hybrid hydrogels assembled under highly crowded conditions (i.e., 25 mg/mL of Ficoll) exhibited similar stiffness to fully covalent‐driven gelatin hydrogels, yet displayed a markedly improved ultimate stress and toughness, effectively overcoming the conventional stiffness–toughness trade‐off in hybrid hydrogels [160]. A similar behavior has been reported for alginate/polyprotein‐based hydrogels, where the introduction of non‐covalent ionic crosslinks significantly enhanced toughness without compromising stiffness [161].

To evaluate the fatigue resistance and energy dissipation behavior of the hybrid hydrogels, five cycles of compressive loading‐unloading tests were conducted. As shown in Figure S11a, all formulations were able to withstand large deformations (up to 65%), with the loading‐unloading curves almost completely overlapping and recovery rates exceeding 97% and 92% after the first and last cycle, respectively (Figure S11b). Although all hydrogels displayed high mechanical resilience, the hysteresis loop (i.e., energy dissipation) of each formulation showed notable differences, particularly while varying the covalent crosslinking density. To better understand this interdependence, the energy dissipation efficiency (EDE) was calculated based on the area of the hysteresis loop and the total area under the unloading curve (Figure 6e) [162]. At the highest TG concentration, all tested formulations displayed a similar EDE when compared to the gelatin control (Figure 6f). We hypothesize that this behavior can be attributed to the higher degree of covalent crosslinking, which limits the contribution of the sacrificial non‐covalent bonds, making the dissipative mechanism predominantly governed by the disruption of covalent crosslinks. However, as the TG concentration decreases, the contribution of the covalent crosslinking diminishes, enabling the G‐quadruplex supramolecular network to function as a dynamic sacrificial network that enhances EDE, largely surpassing the gelatin control values. In contrast, as observed in other fully covalent crosslinked materials [163, 164], the single‐network gelatin control exhibited a reduced energy dissipation while decreasing the TG concentration. The comparison between the hybrid formulations revealed no significant differences in EDE while varying the MMC concentration at comparable TG conditions.

### 2.7 3D Printing Versatility of the G‐Quadruplex Hydrogel Inks

**FIGURE 7:**
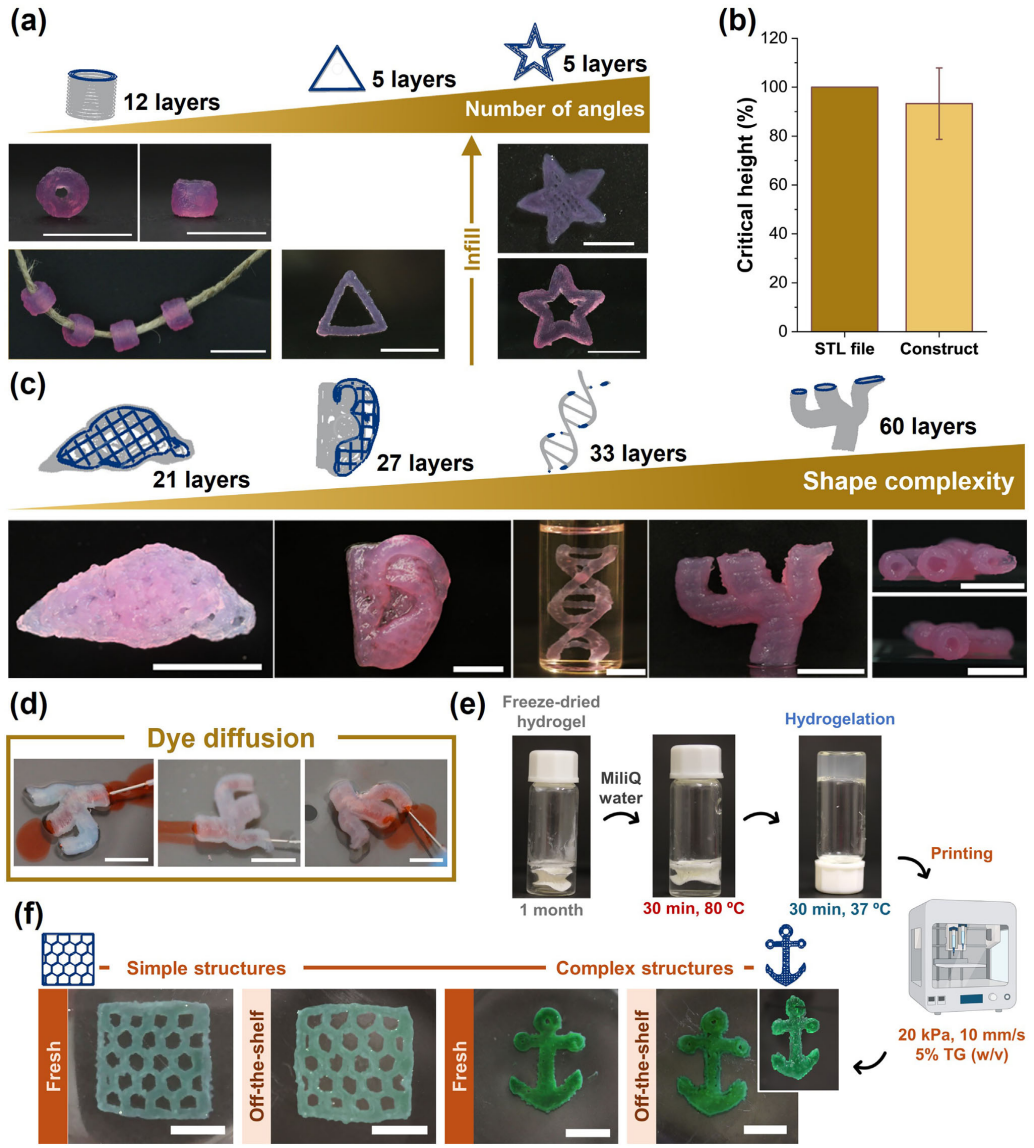
3D Printing versatility of G‐quadruplex hydrogel inks: (a) digital images of 3D printed structures with varying numbers of angles and increasing infill densities; (b) critical height evaluation (*N* = 3); (c) 3D printing of complex and intricated constructs ranging from 21 up to 60 layers; (d) dye diffusion toward the bifurcated outlets of the vascular‐like hollow construct; (e) off‐the‐shelf behavior of the developed ink; (f) 3D printing of grid‐like and anchor‐like structures using both freshly prepared and off‐the‐shelf hydrogel inks. Scale bars: 1 cm. Icon in (e) created with BioRender.com.

Having finely tuned the printing parameters (20 kPa, 10 mm/s) alongside the concentrations of MMC (12.5 mg/mL) and TG (5%, w/v), we then proceeded to demonstrate the capability of the protein‐based MMC‐containing G‐quadruplex ink to faithfully biofabricate intricate, multilayered 3D architectures, from simple to more convoluted ones. We were able to successfully print 5‐ to 12‐layered 3D structures, with increasing infill densities from hollow to almost compact structures, and an increasing number of angles, ranging from tubular (round‐shaped) to star‐shaped geometries (Figure 7a). Additionally, the hollow cylinders were assembled like beads on a string, demonstrating that the constructs could be suspended without breaking or collapsing, highlighting their mechanical robustness and structural integrity. The same structures were used to assess the layer stacking accuracy, defined as the critical height [133] (Figure 7b), demonstrating that increased complexity can be achieved without compromising printing accuracy and closely resembling the stereolithographic designs (93% of critical height achieved). We further tested printing even more complex structures. Figure 7c shows the complexity and intricacy of the printed constructs that range from 21 to 60 layers. Among them, particularly delicate designs such as a DNA coil and a vascular‐like hollow construct with bifurcated outlets were successfully printed. In addition, a dye diffusion assay was attempted, showcasing the potential of the inks to print complex tubular structures that could be used for nerve or vascular tissue engineering applications (Figure 7d; see Video S3). Comparable structural integrity and limited dye diffusion through the walls were reported for an alginate ink crosslinked within the support bath via calcium‐mediated complexation using the well‐established FRESH printing method [165]. Notably, all printed constructs closely matched the original files, clearly demonstrating the ink's ability to accurately reproduce several kinds of patterns and shapes with high fidelity. When compared to previously reported G‐quadruplex‐based inks, our system demonstrates superior performance in terms of printability and shape‐fidelity, especially across complex, multilayered architectures [31, 50, 51, 52]. This improvement arises from the bioinspired hybrid 3D printing strategy proposed here for the first time, in which the synergy between Ficoll‐induced crowding effects and in situ covalent reinforcement significantly enhances both the long‐term stability and printing fidelity. The structural complexity (60 layers) achieved here not only represents a notable advancement over previously reported inks employing the same enzymatic‐mediated in‐bath crosslinking approach [145], but also exceeded the complexity displayed by other protein‐and polysaccharide‐based formulations resorting to alternative in‐bath crosslinking strategies [166, 167, 168, 169].

To further demonstrate the versatility of the developed G‐quadruplex hydrogels, we tested them as an off‐the‐shelf product. The gathered results reveal that the G‐quadruplex hydrogels can be freeze‐dried, stored at room temperature for at least one month, and subsequently rehydrated with Milli‐Q water toward hydrogelation without the need to be refueled with K^+^ ions (Figure 7e). Notably, when compared with the freshly prepared hydrogel ink, the off‐the‐shelf formulation exhibited comparable rheological properties, including oscillatory moduli (Figure S12a) and viscosity (Figure S12b), along with similar G‐quadruplex content, measured by ThT fluorescence assay (Figure S12c). This consistency enabled the ink to retain its printability, using the same crosslinking conditions (5% (w/v) TG) and printing parameters (20 kPa, 10 mm/s), allowing for accurate reproduction of 3D models, from simple square mesh grids to more complex structures, which could be easily handled and detached (Figure 7f). Lastly, the printed grids remained stable for at least 14 days in culture medium at 37°C (Figure S12d), with no significant changes in their area, closely matching the behavior observed for their freshly prepared counterparts. These results demonstrate that the TG‐mediated crosslinking ensures similar long‐term stability in both freshly prepared and off‐the‐shelf constructs. Collectively, these findings enabled us to classify the ink as an off‐the‐shelf product, offering significant convenience for users and broadening its potential clinical applications.

### 2.8 Cell Experiments

#### 2.8.1 Cytocompatibility Assessment of the Hybrid Gelatin‐Based G‐Quadruplex‐Derived Hydrogels

To evaluate the cytocompatibility of the developed hydrogels following the G‐quadruplex assembly and TG‐mediated crosslinking, human adipose‐derived stem cells (hASCs) were seeded on top of the hydrogels, in the absence or in the presence of Ficoll, at concentrations of 5 and 12.5 mg/mL. The cell viability and metabolic activity were assessed over a 7‐day culture period (Figure S13). The live/dead staining revealed high cell viability with no signs of cytotoxicity over the 7‐day culture period for all tested formulations. By day 3, visible cell elongation was observed, followed by pronounced cell spreading and the formation of a confluent cellular layer on the hydrogel surfaces by day 7, particularly in the hydrogel containing 12.5 mg/mL of Ficoll (Figure S13a). Furthermore, the quantification of metabolic activity (by alamarBlue assay) revealed an enhanced cell metabolic activity, as evidenced by a significant increase in fluorescence from day 1 to day 7 across all formulations (Figure S13b). Although no significant differences were observed in the metabolic activity among the formulations by day 7, some differences were noted at days 1 and 3. On day 1, both Ficoll‐containing samples exhibited higher cell metabolic activity when compared to the Ficoll‐free formulation. Interestingly, while in the absence of Ficoll the cell metabolic activity increased sharply from day 1 to day 3, the Ficoll‐containing formulations showed a more gradual and sustained increase throughout the culture period, suggesting a possible positive effect on the inclusion of Ficoll as a protective agent for the initial stress at which cells are submitted. Although this effect is well reported for cell separation and cryopreservation methodologies [111, 170], to the best of our knowledge, this is the first time that is being reported on cytocompatibility assays, given that Ficoll is usually associated with toxicity when used at high concentrations [171, 172].

**FIGURE 8:**
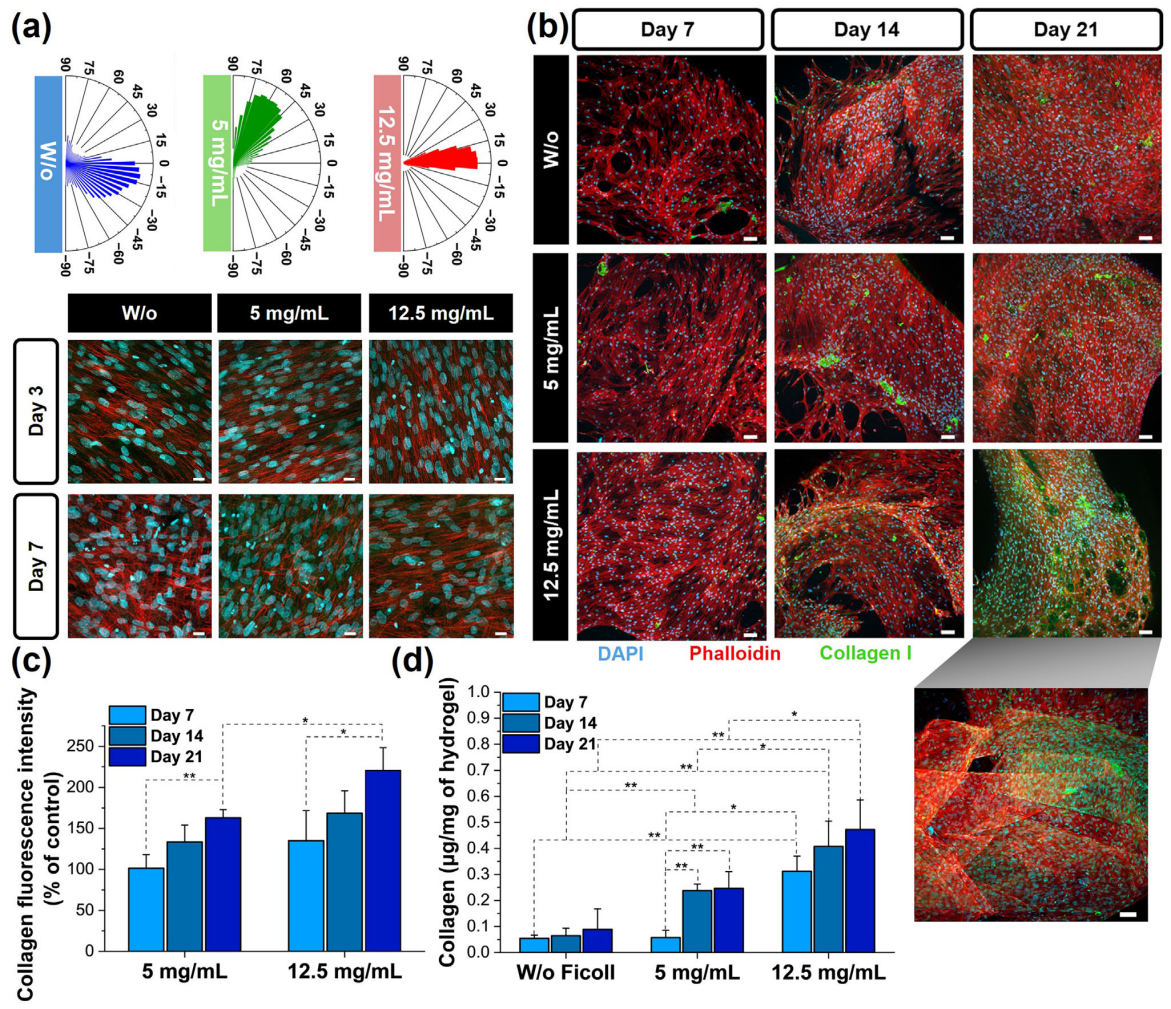
Cell experiments: (a) radial plots of hASCs alignment after 7 days of culture (top). Individual orientation angles were extracted from actin filaments using the Orientation J plugin from ImageJ (Fiji 1.52n). Confocal laser scanning images of DAPI (blue) and phalloidin (red) staining, labeling the nuclei and cytoskeleton, respectively (bottom). Scale bars: 20 µm; (b) confocal laser scanning images of DAPI, phalloidin, and collagen I (green) staining after 7, 14, and 21 days of culture. Scale bars: 100 µm; (c) collagen fluorescence quantification expressed as percentage of the Ficoll‐free control (*N* = 3); (d) total collagen quantification expressed as µg of collagen per mg of scaffold (*N* = 3). Statistical analysis was performed using an unpaired *t*‐test. Individual significance levels are indicated as follows: ^*^ *p* < 0.05, ^**^ *p* < 0.01.

Additionally, phalloidin (red) and DAPI (blue) staining — labeling the cell cytoskeleton and nuclei, respectively — were also performed to evaluate the cell morphology (Figure 8a, bottom). All formulations supported the formation of a confluent and highly interconnected cellular network. To further investigate the cytoskeletal organization, the orientation of actin filaments was analyzed after 7 days of culture [173]. Radial plots analysis revealed a distinct shift in the filament alignment across conditions (Figure 8a, top). In the absence of Ficoll, cells exhibited a disorganized and isotropic actin network, characterized by a broad angular distribution. Conversely, in the presence of Ficoll at 12.5 mg/mL, the actin filaments displayed a highly aligned architecture, reflected by a narrow and sharply defined angular profile. These results suggest that the MMC, by mimicking physiologically confined environments, promotes actin filament alignment and enhances cytoskeletal self‐organization [63]. These early signs of cytoskeletal organization, combined with previous reports showing that macromolecular crowders accelerate the enzymatic conversion of procollagen into collagen [63], prompted us to further investigate the role of macromolecular crowding in collagen deposition. As shown in Figure 8b, all tested formulations displayed signs of collagen I deposition on day 7. However, MMC‐containing formulations showed a consistent increase in collagen staining over time. Interestingly, on day 21, the formation of a collagen‐rich cell sheet on top of the hydrogel containing the highest Ficoll concentration further confirmed the enhanced ECM deposition induced by MMC (Figure 8b, zoom‐in) [174]. We further quantified the collagen immunofluorescence (IF) staining micrographs (Figure 8c). These measurements confirmed the observed trend, with the 12.5 mg/mL formulation yielding the highest percentage of collagen after 21 days of culture. To corroborate IF staining, total collagen quantification was performed on cell‐seeded hydrogels (Figure 8d). This data perfectly matches the amount of collagen stained on the samples, revealing significant differences with both the inclusion and increasing concentration of MMC, being more notorious in later culture days.

Unlike other protein‐free G‐quadruplex‐based hydrogels reported in the literature, which fail to support cell adhesion and consequently lead to cells remaining in a rounded, suspension‐like morphology [50], our hybrid protein‐based G‐quadruplex hydrogel provides important biological cues through intrinsic cell adhesion motifs (e.g., RGD) present in the gelatin backbone, and enhances actin filament alignment due to the crowded environment. This supports robust cell adhesion and spreading, enhanced collagen deposition, and the formation of interconnected cellular networks, representing a significant step toward the development of G‐quadruplex materials with ECM‐mimicking features and enhanced biological relevance. Although the use of MMC to promote actin filament alignment and collagen deposition has been extensively explored across diverse biomedical contexts, ranging from cell culture applications [63, 175] to 3D scaffolds [176, 177], its use to induce collagen deposition on G‐quadruplex‐based hydrogels is, to the best of our knowledge, reported here for the first time.

#### 2.8.2 Bioprinting

**FIGURE 9:**
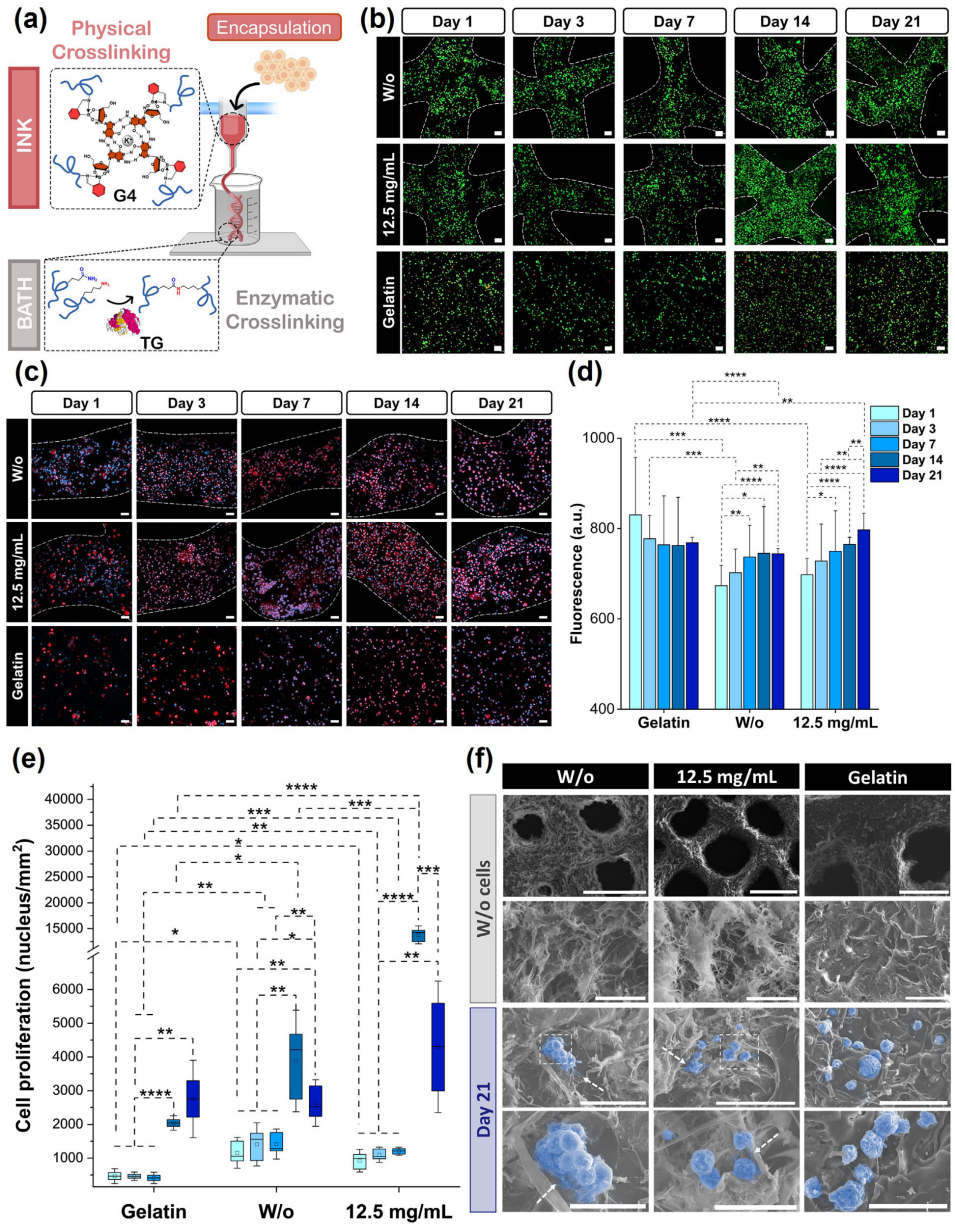
Cell experiments: (a) illustration of the bioprinting set‐up; (b) live/dead fluorescence assay of G‐quadruplex bioinks with and without MMC, using gelatin‐only constructs as a control. Dashed lines indicate the printed filament, which could not be visualized in the gelatin constructs due to poor structural definition. Scale bars: 100 µm; (c) DAPI/phalloidin staining of the 3D printed grids. Scale bars: 50 µm; (d) AlamarBlue assay (*N* = 15 measurements obtained from at least five bioprinted grids); (e) cell proliferation over the 7‐day culture period, determined by quantifying nuclei in DAPI‐stained images (*N* = 3); (f) SEM images of 3D‐printed grids with and without cells after 21 days of culture. Scale bars: 1 mm (top row), 100 µm (middle two rows), and 50 µm (bottom row). Statistical analysis was performed using an unpaired *t*‐test. Individual significance levels are indicated as follows: ^*^ *p* < 0.05, ^**^ *p* < 0.01, ^***^ *p* < 0.001, and ^****^ *p* < 0.0001.

While the cell viability and metabolic activity studies revealed an enhanced actin filament alignment and cytoskeletal organization by the MMC‐containing inks, it was paramount to study these inks as suitable for performing 3D bioprinting, especially the one revealing better cell performance (12.5 mg/mL Ficoll). As such, cells were encapsulated within the ink matrix, bioprinted into 2‐layer mesh grids, and subsequently covalently crosslinked in situ for 2 h at 37°C using enzymatic diffusion inside the support bath (Figure 9a). It is important to note that mass transport in 3D printed constructs is primarily governed by architectural parameters, such as filament diameter, pore size, and interconnectivity, rather than construct height alone, thus justifying the selected 2‐layer square mesh geometry and 10% infill for cellular studies.

It is well‐known that cells can sense and respond to mechanical cues such as matrix stiffness, elasticity, and viscosity [178, 179, 180]. In this context, we first investigated how the hydrogel stiffness (i.e., Young's modulus) would affect either osteosarcoma (Saos‐2) or hASCs cell performance on bioinks, given their known preference for different mechanical properties (Figure S14a–c). For this study, 2.5 and 5% (w/v) of TG were used, since the 1.25% (w/v) formulation, by exhibiting the lowest stiffness, resulted in fragile constructs with limited mechanical integrity, making them unsuitable for cell culture. Cell viability assays revealed that the TG concentration had a cell‐type‐specific response (Figure S14a). While hASCs showed higher viability in the softer 2.5% (w/v) TG crosslinked constructs, Saos‐2 cells exhibited significant cell death under the same condition but showed improved viability when encapsulated in the stiffer 5.0% (w/v) TG crosslinked constructs. The quantitative analysis of the live/dead fluorescence staining further confirmed these results, suggesting distinct preferences for matrix stiffness (Figure S14b,c). Interestingly, these behaviors are consistent with each cell type's native tissue: hASCs originate from soft adipose tissue, whereas Saos‐2 cells are derived from rigid bone tissue. These findings highlight the critical role of fine‐tuning the hydrogel stiffness to better mimic the native biomechanical niche of each specific cell type, which in our case can be achieved by adjusting the TG concentration. The bioprinted constructs further demonstrated to maintain the structural stability, retaining ∼94% of their original area after 21 days of culture, confirming that the mechanical integrity was preserved even under softer conditions (Figure S14d). Following the optimization of the TG concentration on the bioinks, we proceeded to study clinically‐relevant hASCs encapsulated in the gelatin‐based G‐quadruplex‐derived bioink, and monitored their biological performance after 1, 3, 7, 14, and 21 days using gelatin‐only as a control. As shown in Figure 9b, the G‐quadruplex bioink effectively supported high cell viability throughout the 21‐day culture period in both the presence or absence of MMC, with a homogenous cell distribution along the printed filaments and an evident increase in cell number by day 14. In contrast, the gelatin control bioink exhibited reduced cell viability, which can be attributed to the near‐bulk morphology of the printed scaffold, arising from gelatin's limited printability, as previously described. To corroborate these findings, DAPI/phalloidin staining was performed, as it provides a deeper insight into the effects of MMC on cell morphology (Figure 9c). Notably, the incorporation of MMC into the G‐quadruplex bioink enhanced cell clustering. As also supported by the cell seeding assays, we hypothesize that the inclusion of Ficoll enhances extracellular matrix production, positively influencing cell‐cell and cell‐matrix interactions. A similar behavior has already been observed with other crowding agents, where progressive cell clustering occurs [181]. Additionally, another study corroborated our findings by revealing that Ficoll favored cell‐cell interactions, ultimately leading to the stabilization of perfusable microvascular networks [182]. To corroborate our qualitative findings, the cellular metabolic activity was also quantified, revealing that cells encapsulated within the gelatin control showed a decrease in metabolic activity from day 1 to day 21. In contrast, the cells encapsulated in G‐quadruplex bioinks, in particular in the Ficoll‐containing formulation, demonstrated a consistent increase over time, being indicative of a more cytocompatible environment (Figure 9d). Moreover, since significant shear‐induced damage would prevent cells from recovering or proliferating within the first 24 h, and no such effect was observed in early post‐printing viability and metabolic activity assessments, we hypothesize that shear‐related damage plays a negligible role in cell performance. Further quantification of cell proliferation over time revealed a stagnated proliferation in the gelatin control during the first 7 days of culture, followed by a marked increase at days 14 and 21 (Figure 9e). In contrast, G‐quadruplex‐based bioinks exhibited a consistent increase in cell proliferation during the first 7 days, reaching its highest level on day 14 before declining by day 21, particularly for the MMC‐containing formulation.

This late‐stage decline may be attributed to density‐dependent growth regulation mechanisms. We hypothesize that the marked proliferation at day 14 led to high cell density, resulting in crowding, deposition of ECM, and intercellular mechanical compression, which consequently suppressed proliferation [183, 184, 185]. Furthermore, the presence of Ficoll may amplify these effects by promoting additional cell–cell interactions and increasing the overall crowding of the system. Indeed, a similar phenomenon has been previously reported in hASCs cultures supplemented with Ficoll, where the presence of MMC was associated with reduced proliferative capacity [186]. Lastly, after 21 days of culture, the 3D printed mesh grids encapsulating hASCs underwent solvent dehydration followed by critical point drying — a widely used technique to analyze biological specimens [187] — and were subsequently examined by SEM (Figure 9f). SEM analysis showed that the gelatin bioink exhibited a sparse and round‐shaped cell population, which can be indicative of weak cell‐material interactions, possibly due to its smoother surface. In contrast, cells within the G‐quadruplex‐based bioinks appeared well integrated into the matrix, exhibiting longer filopodia (highlighted with white arrows), indicative of cell–cell and cell–matrix interactions, which in turn are facilitated by the hydrogel's fibrillar, ECM‐mimetic topography. This may explain the improved cellular performance observed in the G‐quadruplex constructs when compared to gelatin alone. While previously reported G‐quadruplex hydrogels showed a significant decrease in the viability of immortalized cells after just 24 h of culture [188], our findings highlight the potential of the developed gelatin‐based G‐quadruplex‐derived hydrogel as a promising bioink platform for supporting cell viability and proliferation. This enhanced performance arises from the synergistic interplay between the biofunctionality of gelatin and the fibrillar architecture conferred by the G‐quadruplex, while also providing valuable insights into the influence of the crowded environment on cellular behavior. However, it is noteworthy that, despite the high cell viability and the presence of filopodia observed at extended periods of culture, the cells retained a roundish morphology, corroborating the previous findings reported in other G‐quadruplex‐based bioinks [51] and hydrogels [189]. While lineage‐specific differentiation and the assessment of path dependent mechanisms are critical for translational applications, their evaluation requires tailored biochemical and mechanical cues, which is beyond the scope of this platform‐oriented study. Collectively, our results demonstrate the bioink's versatility and validate its suitability as a foundation for future application‐driven developments. Still, in vivo experiments are needed to undoubtedly make considerations on the biocompatibility of the developed G‐quadruplex hydrogel bioink. Although such studies are out of the scope of this study, previously reported in vivo studies on G‐quadruplex hydrogels have demonstrated their biocompatibility [188], antimicrobial properties, and regenerative capacity, especially in skin/wound healing [24, 190]. Therefore, based on the gathered in vitro data and comparison with similar systems implanted in vivo, the herein developed bioink is expected to hold great promise for tissue engineering and regenerative medicine applications.

## 3 Conclusion

In this work, we developed for the first time a hybrid G‐quadruplex‐derived hydrogel with an increased stability through the synergistic combination of supramolecular self‐assembly, under macromolecular crowding conditions, and enzymatic‐mediated covalent crosslinking. Diluted self‐assembly studies revealed that the incorporation of Ficoll not only triggers a conformational shift from a parallel to an antiparallel G‐quadruplex secondary structure, but also induces the formation of a more densely packed fibrillar network. To promote long‐term stability, an in‐bath enzymatic‐mediated covalent fixation strategy was implemented. Together with macromolecular crowding, this proved to be highly advantageous, effectively overcoming the intrinsic instability that typically limits the biomedical application of G‐quadruplex‐based hydrogels. Notably, besides its importance on the enhanced stability, Ficoll also significantly improved the viscoelastic properties of the ink, namely its viscosity, yield stress, and shear‐thinning profile, being paramount on the printability of a high number of layers (at least 60 layers) with exceptional fidelity. Importantly, the ECM‐mimetic fibrillar nature was kept even after extrusion and subsequently in situ covalent crosslinking, indicating that the supramolecular assembly remains robust, preserving crucial cues essential for enhanced biological performance. Interestingly, we demonstrated the ability to fine‐tune the cellular responses by modulating the TG concentration, underscoring the bioink's versatility for applications across multiple targeted tissues. By providing key insights into how the MMC aids the stability of the supramolecular structures and modulates viscoelastic properties, we envisage that our approach can be translated to other 3D printable supramolecular systems. Lastly, the fact that the supramolecular ink is highly accessible as an off‐the‐shelf biomaterial product unlocks new avenues in advanced biofabrication strategies for tissue engineering and regenerative medicine.

## 4 Experimental Section

### 4.1 Materials

G, agarose, gelatin, Ficoll (MW 400 and 70 kDa), κ‐Carrageenan, and KOH were purchased from Sigma‐Aldrich. KOH solution was prepared using ultrapure water obtained from a Millipore Milli‐Q Plus water purification system (resistivity > 18.2 MΩ·cm). TG and 2‐FPBA were purchased from Cook Lab, Lda., and TCI, respectively. Unless otherwise stated, all chemicals were used as received without further purification.

### 4.2 Determination of NH_2_ Content

The content of free NH_2_ groups on the gelatin backbone was determined through a fast and sensitive assay in which 2,4,6‐trinitrobenzenesulfonic acid (TNBS) reacts with primary amines, forming a chromogenic compound quantifiable at 335 nm using a Synergy HTX multi‐mode microplate reader (BioTek Instruments, Winooski, VT). A calibration curve using glycine standards (0, 1, 5, 15, and 30 µg/mL) was obtained (Figure S1), allowing the determination of the NH_2_ content as 0.202 mmol per gram of gelatin.

### 4.3 Preparation of the Hybrid Gelatin‐Based G‐Quadruplex‐Derived Hydrogels

To prepare the hybrid hydrogels, gelatin (12.8% w/v), G (36 mM), 2‐FPBA (36 mM), and varying concentrations of MMC (0, 5, 12.5, or 25 mg/mL) were added to 1 mL of KOH solution (66 mM, pH 7.6). The precursor mixture was then heated at 80 ºC for 30 min and subsequently cooled to 37°C for an additional 30 min to allow complete hydrogelation. Upon complete gelation, the hydrogel exhibited a pH of 7.1. To further reinforce the hydrogel network, enzymatic‐mediated covalent crosslinking was performed using TG. Briefly, TG was dissolved in an agarose bath at final concentrations of 1.25, 2.5, or 5% (w/v); at this point, the pre‐formed G‐quadruplex supramolecular hydrogels were immersed in the TG‐containing agarose bath and incubated at 37°C for 2 h.

### 4.4 ^1^H NMR Spectroscopy

The ^1^H NMR spectra of gelatin‐based G‐quadruplex‐derived hydrogels, gelatin, G, and 2‐FPBA, were recorded on an Avance II 300 MHz spectrometer (Bruker, Germany) at 300.13 MHz using tetramethylsilane (TMS) as internal reference. To acquire the ^1^H NMR spectrum of the hydrogel, the appropriate amounts of gelatin, G, and 2‐FPBA were dissolved in 1 mL of KOH solution prepared in D_2_O and heated to 80°C. The pH of the KOH solution was adjusted to 7.6 beforehand using deuterium chloride (DCl) and sodium deuteroxide (NaOD). The resulting clear solution was then transferred into an NMR tube and cooled to 37°C to allow hydrogel formation. For comparison, the ^1^H NMR spectra of the control samples (gelatin, G, and 2‐FPBA individually) were also acquired in KOH solution under the same conditions.

### 4.5 ATR‐FTIR Spectroscopy

FTIR spectra of the freeze‐dried hydrogel, gelatin, G, and 2‐FPBA were acquired using a Tensor 27 spectrometer (Bruker, Germany) equipped with a “Golden Gate” ATR module featuring a diamond crystal. Measurements were performed in transmittance mode over the spectral range of 4000–400 cm^−1^, averaging 256 individual scans per sample at a resolution of 4 cm^−1^.

### 4.6 SAXS

Bulk SAXS measurements of the freeze‐dried hydrogel (with and without MMC), as well as gelatin, G, 2‐FPBA, and Ficoll were performed on a Ganesha instrument from SAXSLab. The flight tube and sample holder were all under vacuum in a single housing, with a GeniX‐Cu ultra‐low divergence X‐ray generator. The source produces X‐rays with a wavelength (λ) of 0.154 nm and a flux of 1 × 108 ph s^−1^. Scattered X‐rays were captured on a 2D Pilatus 300K detector with 487 × 619 pixel resolution. The sample‐to‐detector distance was 0.084 m (WAXS mode) or 0.48 m (MAXS mode). The measurement time was 300 s (WAXS mode) or 1200 s (MAXS mode). The instrument was calibrated with diffraction patterns from silver behenate. Samples were taken and inserted in a borosilicate glass capillary with an inner diameter of 1 mm. Domain spacings (d) are calculated using d = 2π/q*, with q* the principal scattering peak.

### 4.7 CD Spectroscopy

The CD spectrum of the freshly prepared gelatin‐based G‐quadruplex‐derived hydrogel, diluted 28‐fold in the presence or absence of MMC, was recorded at 37°C on a J‐1500 CD spectrometer (JASCO, Japan) from 190 to 350 nm at a scan rate of 100 nm/min with a data pitch of 1.0 nm. The CD spectrum was obtained as the average of three accumulations. Baseline correction was performed using a gelatin solution at the same concentration as in the diluted G‐quadruplex samples to remove the influence of the strong negative band from gelatin's random coil conformation, which otherwise masked the G‐quadruplex features in the CD spectra. Additionally, CD spectra of all individual components were also recorded for comparation.

### 4.8 STEM

STEM analysis of the gelatin‐based G‐quadruplex‐derived hydrogel, with or without MMC and diluted 2000‐fold, was carried out using a field emission gun scanning transmission electron microscope (Hitachi STEMHD‐2700, Hitachi High‐Technologies, Japan) operated at an acceleration voltage of 200 kV. For sample preparation, a 5 µL drop of the diluted hydrogel was cast onto a carbon film‐coated copper TEM grids (CF400‐Cu‐carbon film 400 square mesh copper grid) and left to air‐dry overnight at room temperature in a fume hood.

### 4.9 ThT Fluorescence Assay

To confirm the G‐quadruplex formation prior to covalent fixation, 1 mL of ThT‐loaded gelatin‐based G‐quadruplex‐derived hydrogel, with or without MMC, was transferred to a quartz cuvette. Fluorescence spectra were recorded using a FP‐8300 fluorometer (JASCO, Japan), with the emission bandwidth set to 2.5 nm and the data interval to 0.5 nm. Spectra were obtained at λ_ex_ of 450 nm in a data range of 460–750 nm. For comparison, spectra were also recorded for the ThT solution alone (1 mM) and for the precursor mixture lacking K^+^ but containing all other hydrogel components, including Ficoll.

To evaluate the stability of G‐quadruplex structures following covalent fixation with 5% (w/v) of TG, the fluorescence intensity of hydrogels containing varying concentrations of MMC (0, 12.5, and 25 mg/mL) was measured at specific time‐points using a sample holder equipped with a quartz window. Briefly, after physical (G‐quadruplex assembly) and in‐bath chemical (TG‐mediated) crosslinking, the hydrogels were washed in culture medium to remove excess agarose, incubated in a ThT solution (1 mM) for 15 min, and subsequently washed in distilled water for 1 h, with water replacement every 30 min to eliminate unbound ThT and minimize background signal. This procedure was repeated after 1, 3, 7, and 14 days of incubation in culture medium at 37°C. For each hydrogel formulation, fluorescence intensity measured immediately after preparation (day 0) was taken as 100%, enabling the quantification of G‐quadruplex disassembly (%) over time.

### 4.10 Degradation Studies

Hydrogels were prepared as described above and incubated in culture medium at 37°C for several time‐points. At each selected time‐point (30, 60, 180, 360, and 720 min) the hydrogels were removed, freeze‐dried, and their dry weight (D_w_) was measured. In parallel, degradation studies were also conducted after covalent fixation over extended incubation times of 1, 3, 7 and 14 days. The remaining gel weight (%) at each time‐point was calculated according to Equation (1).

(1) $$Hydrogel\;weight\;\left(\%\right)=\frac{D_{w_{time-point}\;}}{D_{w_{t_{0}}\;}}\times 100$$

where $D_{w_{t_{0}}\;}$ is the dry weight immediately after preparation and $D_{w_{time-point}\;}$ is the dry weight at each time‐point.

### 4.11 Water Content

Hydrogels containing varying concentrations of MMC (0, 12.5, and 25 mg/mL) were collected after 1 and 3 days of incubation in culture medium at 37°C, and after carefully removing the excess medium, their wet weight was measured. The samples were then frozen at −80°C, freeze‐dried, and their dry weight determined. The water content (%) was subsequently calculated as previously described [9].

### 4.12 Rheology Measurements

Rheological characterization of the gelatin‐based G‐quadruplex‐derived inks, both without and with 12.5 or 25 mg/mL of Ficoll, was performed before and after covalent reinforcement using a Kinexus Lab+ rotational rheometer (Malvern PANalytical, U.K.) equipped with an 8.0 mm stainless‐steel parallel plate geometry and a fixed gap of 1 mm. All measurements were conducted at 37 °C with a constant frequency of 1 Hz. Oscillatory strain sweeps at 1 Hz were carried out to determine the LVR, and a shear strain of 0.1% — within the LVR — was applied for subsequent tests. To investigate the viscoelastic behavior of the developed inks, oscillatory frequency sweep tests were conducted before and after TG‐mediated crosslinking to measure both the G′ and G′′ moduli, with stiffness assessed as the G′ value at 1 Hz. Additionally, the shear‐thinning behavior of the inks was evaluated by measuring their viscosity as a function of increasing shear‐rate (0.1 to 100 s^−1^). To gain further insight into the ink's 𝜎_0_ and η_0_, shear‐stress ramp assays were performed over a range of 15 to 237 Pa. Next, the self‐healing ability was also assessed by dynamic rheology and employing alternate low (0.1%) and high (100%) strains cycles. The self‐healing efficiency was obtained for the first and last deformation cycle as the ratio between G' value of the healed gel and the original G' value. Moreover, three‐step thixotropic assays were conducted, starting with a low shear‐rate (0.1 s^−1^), followed by a high shear‐rate, and concluding with a recovery phase during which the low shear‐rate was reapplied. The shear‐rate applied in the second step was calculated individually for each ink formulation (Table 1; Equation S1). Lastly, time sweep tests were carried out to evaluate hydrogelation kinetics. All data were collected in triplicates, and the empirical models were fitted using OriginPro software (version 10.1.0.178).

### 4.13 Mechanical Analysis

The mechanical properties of the hybrid hydrogel were evaluated at varying TG concentrations (1.25%, 2.5%, and 5% w/v) using a Universal Mechanical Testing Machine (Shimadzu MMT‐101N, Shimadzu Scientific Instruments, Kyoto, Japan) equipped with a 50 N load cell. Compression tests were performed on freshly hydrated cylindrical samples, each with a diameter of 5 mm and a height of 6 mm, under both unidirectional and cyclic loading conditions. All tests were conducted at room temperature, with a constant compression rate of 1 mm/min for static compression and 5 mm/min for cyclic loading.

Young's modulus was calculated from the slope of the linear portion of the stress–strain curve, within the first 10% of compressive strain. For the cyclic loading‐unloading tests, five cycles were applied at a constant compressive strain of 65%. Recovery percentage was calculated taking the compressive stress of the first cycle as 100%.Toughness was determined as the area under the stress‐strain curve. Energy dissipation efficiency was calculated as described elsewhere [162]. All tests were performed in triplicate.

### 4.14 3D Printing Setup

The gelatin‐based G‐quadruplex‐derived inks were prepared as described above, loaded into a printer cartridge, and printed using a BioX bioprinter (BioX, CELLINK). Briefly, a 22G (410 µm inner diameter) blunt needle was used to print the G‐quadruplex supramolecular ink into an agarose supporting bath containing 5% of TG (w/v), prepared as reported elsewhere [145]. The infill density of the printed grids was set to 15%. All the printing models used to assess the critical height, as well as the complex multilayered constructs, were designed using Shapr3D software (version 5.450) and post‐processed with BioX HeartOS (v2.0, CELLINK). Both the printing head and bed were set at 37°C; post‐printing, all the constructs were incubated 2 h at 37°C.

### 4.15 Printing Fidelity

Filament fidelity was assessed by printing 1 cm‐long filaments at varying printing speeds (8, 9, 10, 12, and 14 mm/s) and pressures (20, 22, and 24 kPa). Filament fidelity was calculated as the ratio between the printed filament diameter and the theoretical nozzle inner diameter (410 µm). The acceptable printability range was defined as values between 0.85 and 1.15 (i.e., diameters between ca. 349 and 472 µm) [191]. Other parameters were also investigated to evaluate the ink's printability. A 2‐layer square mesh grid (L × W × H; 20 mm × 20 mm × 1 mm) was printed and used to determine the mesh printability, internal mesh angle, and area printability. The mesh printability was calculated as previously established [46], using Equation (2). Optical images of the printed grids were analyzed and measured in ImageJ (Fiji 1.52n). For the distribution curves, the N value (*N* = 164) represents the average of the total mesh printability values for the ink without (*N* = 148) and with 12.5 mg/mL of Ficoll (*N* = 180). This difference is attributed to the lower number of printability values obtained for the ink without Ficoll at 14 mm/s, as some of the mesh grids were fragmented and their printability could not be assessed.

(2) $$Mesh\;printability=\frac{L^{2}}{16A}$$

where *L* is the perimeter and *A* is the area of a single mesh square.

### 4.16 Off‐the‐Shelf Approach

The supramolecular inks were prepared as described above, freeze‐dried, and stored at room temperature for at least one month. Afterward, 1 mL of Milli‐Q water was added, and the samples were heated at 80°C for 30 min, followed by cooling to 37°C for 30 min to ensure complete hydrogelation. Finally, the inks were printed using the same printing settings (10 mm/s, 20 kPa) into an agarose bath, following the same crosslinking procedure (5% (w/v) TG, 2 h at 37°C).

### 4.17 SEM

The morphology of the inks pre‐ and post‐printing was analyzed in a scanning electron microscope (SEM Hitachi SU‐3800, Japan) equipped with a standard cooling stage (Deben, UK), which allows temperature control between −25°C and 50°C. The microscope was operated in secondary electron mode at an acceleration voltage of 10 kV. Prior to imaging, samples were deposited on a metal stub containing a cryogenic embedding medium (Tissue‐Tek O.C.T compound), and image acquisition was carried out at 37°C.

### 4.18 Cell Culture

Human adipose‐derived stem cells (hASCs; ATCC PCS‐500‐011) and the Saos‐2 cell line (ECACC, Sigma–Aldrich) were cultured in T‐175 tissue culture flasks (Sarstedt) using α‐MEM (ThermoFisher Scientific) and low‐glucose Dulbecco's Modified Eagle Medium (DMEM; Sigma–Aldrich), respectively. Both media were supplemented with 10% fetal bovine serum (FBS) and 1% antibiotic–antimycotic solution, and cells were maintained under standard culture conditions (37°C, 5% CO_2_).

### 4.19 Seeding Assays

hASCs were seeded on top of the bulk hybrid G‐quadruplex hydrogels, previously washed in culture medium for 24 h to remove residual agarose, at a density of 3 × 10^4^ cells per gel. Cell‐seeded hydrogels were cultured for 7 days, and the medium was changed every 2–3 days.

### 4.20 Printing of Cell‐Laden Grids

For preliminary assays, once confluence was reached, both Saos‐2 cells and hASCs were trypsinized and encapsulated in fully gelated bioinks at a density of 9 × 10^6^ cells per mL. After ensuring homogeneity by gentle up‐and‐down pipetting, the cell‐laden bioinks were loaded into sterile cartridges and used to print a 2‐layer square mesh grid (L × W × H: 10 mm × 10 mm × 1 mm; 10% infill) into sterile 12‐well plates pre‐filled with 1 mL of TG‐containing agarose support bath. This reduced construct size was chosen to facilitate imaging.

Following TG concentration optimization, the hASCs cell density was raised to 15 × 10^6^ cells per mL, and the same mesh geometry was printed and covalently crosslinked with 2.5% (w/v) of TG for 2 h at 37°C. All constructs were cultured for up to 7 days, with specific time‐points designated for cellular performance evaluation. A printing pressure of 20 kPa was applied for all bioprinting experiments. To minimize the shear‐rate experienced by cells during extrusion, the printing speed was reduced to 8 mm/s. For all 3D bioprinting experiments, both the printing head and bed were maintained at 37°C. Gelatin‐only bioinks (matching the gelatin concentration used in the G‐quadruplex formulations) were employed as controls. These were subjected to the same heating–cooling procedure and printed at 5 kPa and 10 mm/s, following conditions previously reported [145]. Although these settings did not fully preserve the pore geometry, they were selected as the most suitable compromise to prevent complete collapse of the printed structure into a bulk form, given the low viscosity of gelatin at 37°C.

### 4.21 Cell Viability Assays

At each time‐point (1, 3, 7, 14, and 21 days), cell viability was analyzed using a Live/Dead staining assay. Briefly, samples were incubated at 37°C for 30 min in Dulbecco's Phosphate‐Buffered Saline (DPBS; Sigma‐Aldrich) containing 2 µg/mL of calcein‐AM (ThermoFisher Scientific) and 1 µg/mL of propidium iodide (PI, ThermoFisher Scientific). Cell‐laden bioinks were imaged using a confocal laser scanning microscope (CLSM 900, Carl Zeiss Microscopy, Germany), while cells seeded on bulk hydrogels were visualized with a Zeiss Axio Imager M2 upright fluorescence microscope (Carl Zeiss, Germany). Image analysis was performed using ZEN Blue Edition v2.3 software (Zeiss). Metabolic activity was assessed using the AlamarBlue Cell Viability Reagent (Alfagene, Lisbon, Portugal). At predetermined time‐points, the culture medium was removed and a 10% (w/v) AlamarBlue solution prepared in α‐MEM was added to the samples and incubated for 4 h at 37°C. Fluorescence measurements were performed in a Synergy HTX multi‐mode microplate reader (BioTek Instruments, Winooski, VT) with excitation and emission wavelengths of 540/35 nm and 600/40 nm, respectively.

### 4.22 Cell morphology evaluation

To analyze cell morphology, samples were first incubated with DAPI (1:1000 v/v in DPBS, Thermo Fisher Scientific) for 30 min at 37 °C, washed twice with DPBS, and subsequently incubated with phalloidin (1:40 v/v in DPBS, Flash Phalloidin Red 594, BioLegend) for an additional 30 min at 37°C, followed by two final DPBS washes. Prior to staining, cell‐laden constructs were permeabilized for 30 min at 37°C under constant agitation using a 0.1% (v/v) Triton X‐100 solution (Triton X‐100 BioXtra, Sigma‐Aldrich) prepared in DPBS. Stained, cell‐laden 3D printed constructs and cell‐seeded bulk hydrogels were imaged using a confocal and widefield fluorescence microscope, respectively. Cell proliferation was evaluated by quantifying nuclei in DAPI‐stained images, with cell density (cell/mm^2^) determined using ImageJ (Fiji 1.52n).

### 4.23 Immunofluorescence Collagen Labeling

To analyze collagen deposition, cell‐seeded hydrogels were fixed with 4% paraformaldehyde and permeabilized with 0.1% Triton X‐100 for 30 min at 37°C under constant agitation. The samples were then blocked with 3% bovine serum albumin (BSA) for 1 h at 37°C. Subsequently, the scaffolds were incubated overnight at 4°C with a mouse monoclonal anti‐collagen I antibody (1:100 dilution in 3% BSA, Thermo Fisher Scientific). After three washes with DPBS, samples were incubated for 2 h at 37°C with Alexa Fluor 647 goat anti‐mouse IgG secondary antibody (1:500 dilution in 3% BSA, Alfagene). Following additional washes with DPBS, nuclei and F‐actin were stained with DAPI and phalloidin, respectively, as described above. Imaging was performed using a confocal laser scanning microscope. Collagen fluorescence intensity was quantified using ImageJ software, and results were expressed as a percentage relative to the Ficoll‐free cell‐seeded hydrogel control.

### 4.24 Total Collagen Assay

To quantify collagen content, a total collagen assay kit was used (AkrivisBio). Briefly, the collagen standard was first hydrolyzed at 120°C for 1 h with 10 M NaOH. After cooling on ice, the solution was neutralized with 10 M HCl, centrifuged at 10 000 × *g* for 5 min, and aliquots of 0, 2, 4, 6, 8, and 10 µL of the 1 mg/mL hydrolyzed standard were added to the wells, corresponding to 0, 2, 4, 6, 8, and 10 µg of collagen, respectively. Then, the standards were evaporated to dryness by heating the plate to 65–80°C. For the cell‐seeded hydrogels (after 7, 14, and 21 days of culture), each sample was weighed, and deionized water was added at a ratio of 100 µL per 10 mg of hydrogel. An equal volume of 10 M NaOH was then added, and the samples were hydrolyzed at 120°C for 1 h, neutralized with 10 M HCl, and centrifuged to pellet insoluble debris. Then, 10 µL of each neutralized hydrolysate were transferred to a 96‐well plate and evaporated to dryness by heating the plate to 65–80°C. For both standards and samples, 100 µL of oxidation mixture (6 µL Chloramine T Concentrate + 94 µL Oxidation Buffer) was added to each well and incubated at room temperature for 20 min. Next, 100 µL of DMAB reagent (prepared by mixing 50 µL DMAB Concentrate with 50 µL Acid/Isopropanol Solution) was added, and the plate was incubated at 65°C for 45 min. Optical density was measured at 560 nm. Collagen content was calculated using the standard curve (Figure S13c) and is presented as µg of collagen per mg of scaffold. Cell‐free scaffolds were used as blank controls.

### 4.25 SEM Analysis of the Cell‐Laden Grids

Cell‐laden grids, after 21 days of culture, were dehydrated through a graded ethanol series (50%, 70%, 80%, and 95%, for 10 min each), followed by two 15‐min incubations in absolute ethanol. Samples were then placed into microporous specimen capsules, which were subsequently immersed in absolute ethanol overnight. Finally, samples were dried using a critical point dryer, fixed onto aluminum stubs using double‐sided carbon conductive adhesive tape, and sputter‐coated with carbon (K950X Turbo Evaporator, Emitech, U.S.).

### 4.26 Statistical Analysis

All data were statistically evaluated resorting to GraphPad Prism software (version 10.1.0) and reported as mean ± standard deviation. Statistical significance was assessed using an unpaired *t*‐test, with differences considered significant at *p* < 0.05. To compare filament printability values among different pressures, normality was checked using the Shapiro–Wilk test. Subsequently, a Kruskal–Wallis test followed by Dunn's post hoc test was employed.

## Conflicts of Interest

The authors declare no conflicts of interest.

## Supporting information




**Supporting File 1**: adma72984‐sup‐0001‐SuppMat.docx.

**Supporting File 2**: adma72984‐sup‐0002‐VideoS1.mp4.

**Supporting File 3**: adma72984‐sup‐0003‐VideoS2.mp4.

**Supporting File 4**: adma72984‐sup‐0004‐VideoS3.mp4.

## Data Availability

The data that support the findings of this study are available from the corresponding author upon reasonable request.
