# Supplementary material for: Stable Protein‐Based G‐Quadruplex‐Derived Supramolecular Bioinks as Tunable ECM‐Mimetic Constructs Assembled by Combining Non‐Covalent and Covalent Strategies
Source: Adv Mater. 2026 Apr 8;38(44):e21259. doi: 10.1002/adma.202521259 (PMC13449135; doi:10.1002/adma.202521259)
Supplement: Supplementary file 1 [file ADMA-38-e21259-s004.docx]

**Supporting Information**

**Stable Protein-Based G-Quadruplex-Derived Supramolecular Bioinks as Tunable ECM-Mimetic Constructs Assembled By Combining Non-Covalent and Covalent Strategies**

Vera Sousa,^1^ Rita Sobreiro-Almeida,^1^ Bart W. L. van den Bersselaar,^2,3^ E. W. Meijer,^2,3^ Ghislaine Vantomme,^2,3^ João Borges,^1^* and João F. Mano^1^*

^1^ CICECO – Aveiro Institute of Materials, Department of Chemistry, University of Aveiro, Campus Universitário de Santiago, 3810-193 Aveiro, Portugal

^2^ Institute for Complex Molecular Systems, Eindhoven University of Technology, 5600 MB Eindhoven, The Netherlands

^3^ Department of Chemical Engineering and Chemistry, Laboratory of Macromolecular and Organic Chemistry, Eindhoven University of Technology, 5600 MB Eindhoven, The Netherlands

**Table of contents:**

[1. Formation of the G-quadruplex supramolecular hydrogels	3](#_Toc223349481)

[2. Role of macromolecular crowding on G-quadruplex self-assembly	4](#_Toc223349482)

[3. Synergistic effect of Ficoll-induced dehydration and enzymatic-mediated covalent crosslinking on G-quadruplex stability	6](#_Toc223349483)

[4. Rheological characterization of the G-quadruplex supramolecular hydrogel inks..	6](#_Toc223349484)

[4.1. Effect of macromolecular crowding on ink gelation and flow properties	6](#_Toc223349485)

[5. Screening the filament, mesh and shape-fidelity of the G-quadruplex hydrogel inks	7](#_Toc223349486)

[6. Unveiling the role of in-bath TG-mediated covalent fixation	9](#_Toc223349487)

[7. 3D Printing versatility of the G-quadruplex hydrogel inks	11](#_Toc223349488)

[8. Cell experiments	12](#_Toc223349489)

[8.1. Cytocompatibility assessment of the hybrid gelatin-based G-quadruplex-derived hydrogels	12](#_Toc223349490)

[8.2. Bioprinting	13](#_Toc223349491)

1. Formation of the G-quadruplex supramolecular hydrogels

**Figure S1.** Calibration curve obtained from glycine standards (0, 1, 5,15 and 30 µg/mL) used for the quantification of the free NH_2_ groups in gelatin backbone.

**Figure S2.** (a) ^1^H NMR spectra of gelatin (blue line), guanosine (red line), 2-formylphenylboronic acid (grey line), and the G-quadruplex hydrogel (green line) at 37 ºC in D_2_O at pH = 7.6; (b) ^1^H NMR spectrum of the hydrogel recorded at 25 ºC for comparison.

At 25 °C, gelatin adopts a helical collagen-like structure,^[1]^ which reduces the mobility of the gelatin chains,^[2]^ thereby decreasing the overall spectral resolution and hindering the boronate ester bond signal **(Figure S2b)**. In contrast, at 37 °C, gelatin assumes a random coil conformation,^[1]^ allowing its amino acid side chains to be surrounded by deuterated water molecules,^[3]^ which facilitates the detection of the boronate ester bond peak. It has already been reported that the intensity of the peak assigned to boronate ester bonds increases with increasing temperatures due to the release of more reacted G as the hydrogel melts.^[4]^ However, in this case, the observed behavior is primarily driven by the conformational changes of the gelatin chains.

**Figure S3.** (a) ATR-FTIR spectra of G-quadruplex hydrogel and respective controls; (b) ATR-FTIR spectrum of a mixture containing 2-FPBA, gelatin, and K^+^, but without guanosine (orange line), was also recorded for comparison.

2. Role of macromolecular crowding on G-quadruplex self-assembly

Ficoll is a neutral-charge MMC, which prevents it from creating electrostatic interactions with other molecules, allowing the excluded volume effects to occur without external interference.^[5]^ Given that Ficoll is a synthetic polymer of sucrose and sucrose monomers contain diols, it is also important to confirm whether additional crosslinking between 2-FPBA and the MMC could occur. As shown in **Figure S4a**, hydrogel formation did not occur, indicating no additional crosslinking between the MMC and 2-FPBA. This can be explained by the following general binding affinity of boronic acids to diols in saccharides: *cis*-1,2-diol > 1,3-diol >> *trans*-1,2-diol.^[6]^ Guanosine contains *cis*-1,2-diols, whereas sucrose only contains *trans*-1,2-diols.^[6]^ Therefore, 2-FPBA preferentially reacts with guanosine, which explains the absence of gelation even in the presence of Ficoll.

**Figure S4.** (a) Optical image of a control in which guanosine was removed while all other components were maintained and (b) degradation studies (N = 3). Statistical significance was performed using an unpaired t-test. Individual significance levels are indicated as follows: **p* < 0.05, ***p* < 0.01, ****p* < 0.001. Green, red, and black significance levels correspond to Ficoll_400_ *vs.* without Ficoll, Ficoll_70_ *vs.* without Ficoll, and Ficoll_400_ *vs.* Ficoll_70_, respectively.

**Figure S5.** (a) CD spectrum; (b) MAXS/WAXS patterns of the controls and (c) Z-Contrast-STEM mode images of samples in the diluted state — gelatin only, without Ficoll, and with Ficoll. Scale bars: 3 µm.

3. Synergistic effect of Ficoll-induced dehydration and enzymatic-mediated covalent crosslinking on G-quadruplex stability

**Figure S6.** (a) Optical images of ThT-incubated gelatin and G-quadruplex hydrogel after TG-mediated crosslinking under 365 nm UV light; (b) time-dependent fluorescence profile across all tested hybrid G-quadruplex hydrogel formulations.

4. Rheological characterization of the G-quadruplex supramolecular hydrogel inks

4.1. Effect of macromolecular crowding on ink gelation and flow properties

Equation 1. The shear-rate at the wall (𝛾̇_wall_) can be calculated from the flow rate (Q), which is the product of the nozzle area (πR^2^) and the printing speed (ν). The degree of shear-thinning also influences 𝛾̇_wall_, with lower *n* values resulting in higher 𝛾̇_wall_ values.^[7]^

**Table S1.** Detailed parameters of the models employed in this study. **η (γ̇)** = shear-viscosity (Pa.s); ***n*** = flow index; **K** = consistency index; **γ̇** = shear-rate (s^-1^); **𝜎** = shear-stress (Pa); **𝜎_0_** = yield stress (Pa); **|η* (𝜔)|** = complex viscosity (Pa.s); **𝜔** = angular frequency (rad/s); **η_0_** = zero-shear-viscosity (Pa.s); **η_∞_**= infinite viscosity (Pa.s); **k** and **m**: fitting parameters of the cross model.

Table S2. Comparison of model fitting values of 𝜎_0_ and η_0_ with experimental data, including the viscosity drop taken from shear-stress ramp assays.

5. Screening the filament, mesh and shape-fidelity of the G-quadruplex hydrogel inks

Figure S7. (a) Shear-thinning behavior of the agarose supporting bath before and after TG incorporation; (b) thixotropic behavior of the supporting bath after TG inclusion.

Figure S8. Stereomicroscopic images of the printed filaments at a constant pressure of (a) 22 kPa and (b) 24 kPa; filament diameter evaluation at (c) 22 kPa and (d) 24 kPa (N ≥ 39 measurements per speed obtained from at least six printed filaments); filament printability of the ink without (e) and with 25 mg/mL of Ficoll (f) at different printing pressures as a function of printing speed, with the acceptable printability region highlighted in grey (for 20 kPa, N ≥ 50; for 22 kPa, N ≥ 39; for 24 kPa, N ≥ 39 measurements per speed obtained from at least six printed filaments); (g) pore area (%), calculated as the ratio between the total pore area and the total area of the printed grids, measured at 20 kPa and 10 mm/s, both after printing and after 48h of incubation in culture medium (N = 3). Scale bars: 1 cm. Statistical analysis was performed using a Kruskal-Wallis test followed by Dunn’s post hoc test for (c) and (d), and an unpaired t-test for (g). Individual significance levels are indicated as follows: **p* < 0.05, ***p* < 0.01, ****p* < 0.001, and *****p* < 0.0001.

6. Unveiling the role of in-bath TG-mediated covalent fixation

Figure S9. Frequency sweep assays of the G-quadruplex inks (a) and the gelatin control (b) before and after covalent crosslinking; (c) top and side view of a 5-layer hollow star printed at 37 ºC with a gelatin solution at the same concentration used for the gelatin-based G-quadruplex inks, followed by in-bath covalent fixation with 5% (w/v) of TG; (d) stiffness evaluation before and after covalent crosslinking (N = 3). Statistical analysis was performed using an unpaired t-test for (g). Individual significance levels are indicated as follows: **p* < 0.05, **p < 0.01, *****p* < 0.0001.

Figure S10. Evaluation of (a) Young’s modulus, (b) ultimate strain, and (c) ultimate stress (N = 3). Statistical analysis was performed using an unpaired t-test. Individual significance levels are indicated as follows: **p* < 0.05, ***p* < 0.01, and ****p* < 0.001.

Figure S11. (a) Cyclic loading/unloading compressive curves for all tested formulations at 65% compressive strain; (b) recovery percentages for both the 1^st^ and 5^th^ cycles (N = 3). Statistical analysis was performed using an unpaired t-test. Individual significance levels are indicated as follows: **p* < 0.05 and ***p* < 0.01.

7. 3D Printing versatility of the G-quadruplex hydrogel inks

Figure S12. (a) Frequency sweep assay of both off-the-shelf and freshly prepared inks, with the corresponding stiffness values taken as the G' at 1 Hz (inset, N = 3); (b) complex viscosity as a function of angular frequency, fitted with the Cross model to determine the zero-shear-viscosity (ZSV) of the tested inks (inset, N = 3); (c) ThT fluorescence assay; (d) digital images illustrating the stability of the printed grids in culture medium at 37 °C (N = 3). Statistical analysis was performed using an unpaired t-test, with differences considered significant at *p* < 0.05.

8. Cell experiments

8.1. Cytocompatibility assessment of the hybrid gelatin-based G-quadruplex-derived hydrogels

Figure S13. (a) Live/dead fluorescence microscopy of hASCs seeded on top of the hybrid gelatin-based G-quadruplex-derived hydrogels. Live cells are stained with calcein-AM in green and dead cells are stained with PI in red. Scale bars are 200 µm; (b) metabolic activity assay demonstrated by alamarBlue fluorescence over the 7-day culture period (N = 4); (c) calibration curve used for collagen quantification. Statistical analysis was performed using an unpaired t-test. Individual significance levels are indicated as follows: **p* < 0.05, ***p* < 0.01, ****p* < 0.001, and *****p* < 0.0001.

8.2. Bioprinting

Figure S14. (a) Live/dead staining of hASCs and Saos-2 encapsulated in grid-like constructs crosslinked with 2.5 and 5% (w/v) of TG after 3 days of culture. Scale bars: 200 µm; quantification of cell viability for hASCs (b) and Saos-2 (c) using ImageJ (N = 3); (d) structural stability of the bioprinted grids covalently reinforced with 2.5% (w/v) of TG after 1, 14 and 21 days of culture (N = 3). Scale bars: 1 cm.

References

[1]	F. Polyak, G. Reich, *Eur. J. Pharm. Biopharm.* **2019**, 140, 11.

[2]	M. Zandi, H. Mirzadeh, C. Mayer, *Eur. Polym. J.* **2007**, 43, 1480.

[3]	V. V. Rodin, V. N. Izmailova, *Colloids Surf. A: Physicochem. Eng. Asp.* **1996**, 106, 95.

[4]	H. Qiao, J. Bai, S. Zhang, C. Li, *RSC Adv.* **2020**, 10, 28536.

[5]	A. S. Zeiger, F. C. Loe, R. Li, M. Raghunath, K. J. Van Vliet, *PloS one* **2012**, 7, e37904.

[6]	M. Jiang, A. N. Chattopadhyay, C. H. Li, Y. Geng, D. C. Luther, R. Huang, V. M. Rotello, *Chem. Sci.* **2022**, 13, 12899.

[7]	S. Hafeez, A. A. Aldana, H. Duimel, F. A. A. Ruiter, M. C. Decarli, V. Lapointe, C. van Blitterswijk, L. Moroni, M. B. Baker, *Adv. Mater.* **2023**, 35, 2207053.
